# Identification of novel superoxide dismutase isoenzymes in the olive (*Olea europaea* L.) pollen

**DOI:** 10.1186/s12870-018-1328-z

**Published:** 2018-06-08

**Authors:** Adoración Zafra, Antonio Jesús Castro, Juan de Dios Alché

**Affiliations:** Plant Reproductive Biology Research Laboratory, Estación Experimental del Zaidín, Consejo Superior de Investigaciones Científicas (CSIC), Department of Biochemistry, Cell and Molecular Biology of Plants, Profesor Albareda 1, 18008 Granada, Spain

**Keywords:** Alternative splicing, Amyloplast, Cu,Zn-SOD, Fe-SOD, Mn-SOD, Olive, Peroxisome, Pollen

## Abstract

**Background:**

Among antioxidant enzymes, the superoxide dismutase (SOD) family is a major actor in catalysing the disproportionation of superoxide. Apart from its role as antioxidant, these enzymes have a role in cell signalling, and Cu,Zn-SOD proteins are also major pollen allergens. In order to deepen our understanding of the SOD isoenzymes present in olive pollen and to analyse the molecular variability of the pollen Cu,Zn-SOD family, we carried out biochemical, transcriptomic and localization studies of pollen grains from different olive cultivars and other allergenic species.

**Results:**

Olive pollen showed a high rate of total SOD activity in all cultivars assayed, which did not correlate with pollen viability. Mass spectrometry analysis together with activity assays and Western blotting experiments enabled us to identify new forms of Cu,Zn-SOD enzyme (including chloroplastidic and peroxisomal forms) as well as differentially expressed Mn-, Fe- and Cu,Zn-SOD isoenzymes among the pollen of different olive cultivars and allergenic species. Ultrastructural localization of Cu,Zn-SOD revealed its plastidial localization in the pollen grain. We also identified the occurrence of a shorter form of one of the cytosolic Cu,Zn-SOD enzymes, likely as the result of alternative splicing. This shorter enzyme showed lower SOD activity as compared to the full length form.

**Conclusions:**

The presence of multiple SOD isoenzymes in the olive pollen could be related to the need of finely tuning the ROS metabolism during the transition from its quiescent condition at maturity to a highly metabolically active state at germination.

**Electronic supplementary material:**

The online version of this article (10.1186/s12870-018-1328-z) contains supplementary material, which is available to authorized users.

## Background

Approximately 1% of O_2_ consumed by plants is diverted to produce reactive oxygen species (ROS) at various subcellular loci [1]. Reactive oxygen species is a term that encompasses the hydroxyl radical (HO^.^), hydrogen peroxide (H_2_O_2_), the superoxide radical (O_2_^.-^) and singlet oxygen (^1^O_2_). In the plant kingdom, while ROS production and ROS-induced damage occur under biotic and abiotic stress conditions, ROS are also associated with molecular signalling [2–5]. In higher plants, enhanced oxidation is also a signal for appropriate adjustments in gene expression and cell structure in response to environmental and developmental cues [6].

There is a close relationship between ROS (particularly H_2_O_2_) and plant reproductive biology. Pollen tube growth has high-energy requirements, and ROS result from aerobic metabolism. Furthermore, pollen tube growth is guided through the pistil thanks to the interchange of signals between pollen and pistil tissues, a process in which ROS appear to participate as signals. Reactive oxygen species, mainly H_2_O_2_, are constitutively accumulated in stigmas, and high levels of peroxidase activity have been detected when mature stigmas are receptive to pollen grains [7]. Their accumulation in stigmas is related to nitric oxide (NO) production in pollen grains [8], which seems to negatively modulate H_2_O_2_ when pollen grains stick to stigmatic papillae [7–9]. Moreover, a clear association has been established between ROS and the oscillatory cycles of pollen tube growth in lily [10].

Levels of ROS in cells are controlled by a vast gene network [11]. These ROS are produced during metabolic processes and stress conditions, and they are decreased by a broad antioxidant system. Antioxidants can be non-enzymatic, including ascorbic acid, tocopherol, glutathione, flavonoids, alkaloids and carotenoids [12]. The enzymatic antioxidant system is composed of superoxide dismutase (SOD), catalase (CAT), peroxidase (POD) and the ascorbate-glutathione cycle enzymes [13], among others.

The SOD family catalyses the disproportionation of superoxide (O_2_^**·-**^) radicals in biological systems to form H_2_O_2_ and O_2_ [14], which plays an important role in protecting cells against the toxic effects of superoxide radicals produced in different cellular compartments. Plant SODs are metalloenzymes containing Fe, Mn or Cu/Zn as prosthetic group. The number, type and distribution of SOD isoenzymes can change depending on the species, developmental stage and environmental conditions [15–19].

Iron-SODs, likely the oldest group of SODs [18], are ubiquitous enzymes in plants [20], being inactivated by H_2_O_2_ but resistant to KCN inhibition. A first group of Fe-SODs consists of a homodimer formed by two identical 20 kDa subunits, with one or two Fe atoms in the active centre. A second Fe-SOD group, found in most higher plants, is a tetramer of 80–90 kDa and contains two to four Fe atoms in the active centre [18]. Fe-SODs have been found mainly in chloroplasts [21], and more rarely in mitochondria and peroxisomes [22, 23].

Manganese-SODs are functional homodimers and homotetramers composed of 23 kDa subunits [24]. They are not inhibited by cyanide or H_2_O_2_, being present in mitochondria and peroxisomes [16, 25–27]. The Mn-SOD gene is expressed in all cell types and is the only SOD present in vascular tissues [19].

Copper,Zinc-SODs are ubiquitous and very stable homodimeric enzymes of about 32 kDa [28]. Each subunit contains a Cu atom involved in the dismutation reaction, and a Zn atom that stabilizes the enzyme in order to maintain an appropriate level of catalytic activity and to catalyse the folding of SOD in the physiological buffer [28–30]. This enzyme is reversibly inhibited by cyanide and H_2_O_2_ at concentrations ≥10 μM [31]. Copper/zinc-SODs are located in the cytosol, chloroplasts, peroxisomes and the apoplast [32–39]. Chloroplast Cu,Zn-SOD sequences contain 153 amino acids, with the terminal one being Leu, Ile or Val, while cytosolic isoenzymes are 152 amino acids long, with the last one always being a Gly [40].

Previous work reported up to four Cu,Zn-SOD forms of ~ 16 kDa and p*I*s ranged from 5.1 to 6.5 in the olive pollen, which localized in the cytoplasm of both the vegetative and generative cells [41] and in peroxisomes [39]. Olive pollen Cu,Zn-SOD is also an allergen, with an incidence in the population of 35% [42]. Molecular variability of this allergen in olive pollen was assessed by bioinformatics approaches using an olive pollen transcriptome and further PCR validation [43]. In the present study, we gain a deeper understanding of the different SOD isoenzymes present in olive pollen and reveal the presence of new SOD forms. We also analyse the molecular variability of Cu,Zn-SODs in several cultivars and discuss the importance of this variability in relation to reproductive biology and allergy.

## Results

### Superoxide dismutase activity in the olive pollen grain

**Fig. 1:**
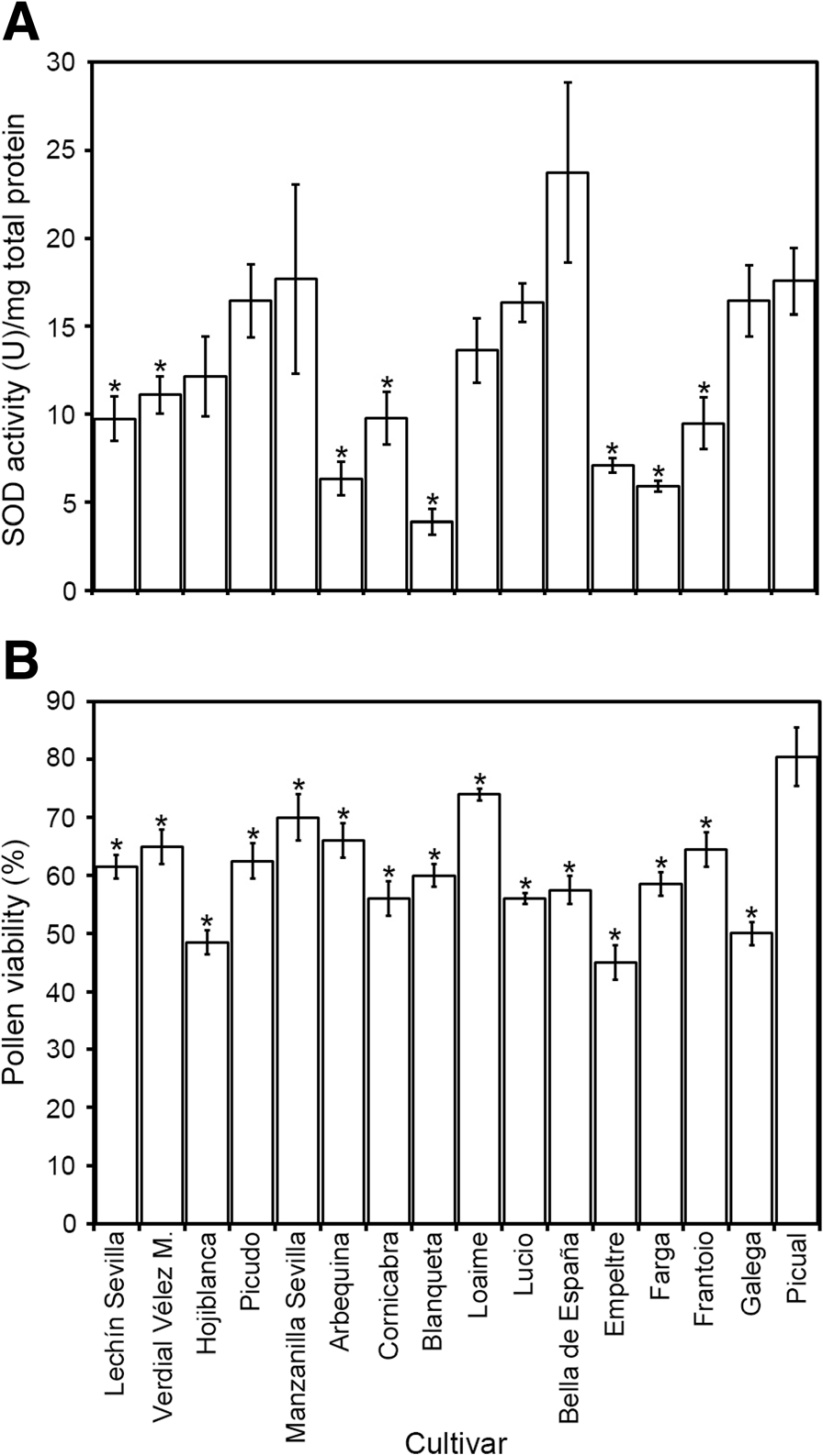
Superoxide dismutase (SOD) activity and viability assays of pollen samples from 16 olive cultivars. **a** Total SOD activity [units (U)/mg of total protein] in pollen protein extracts. **b** Pollen viability assays of the same samples. Cultivar names are shown in the lower chart. The asterisks indicate a significant difference from the cv. ‘Picual’ according to Student’s t test at *p* < 0.01

SOD activity in pollen was higher as compared to other olive tissues such as leaves, which showed rates of 1.7 U/mg protein [19]. Olive pollen protein extracts displayed very different total SOD activities depending on the cultivar (F = 26.648, *p* = 0.000). Thus, total SOD activity ranged from 3.9 (cv. ‘Blanqueta’) to 23.7 (‘Bella de España’) U/mg of total protein (Fig. 1a). Taking ‘Picual’ as the reference cultivar, eight other varieties (‘Lechín’, ‘Verdial’, ‘Arbequina’, ‘Cornicabra’, ‘Blanqueta’, ‘Empeltre’, ‘Farga’ and ‘Frantoio’) showed significant lower activity levels, while the remaining cultivars tested showed similar values (Fig. 1a). Given that pollen viability may change depending on the cultivar and environmental conditions, we also detected differences in this parameter among cultivars (F = 46.868, *p* = 0.000). All the cultivars analysed showed lower viability values compared with ‘Picual’ (Fig. 1b). However, no correlation was found when comparing total SOD activity and pollen viability (*r* = 0.177, *p* = 0.228).

**Fig. 2:**
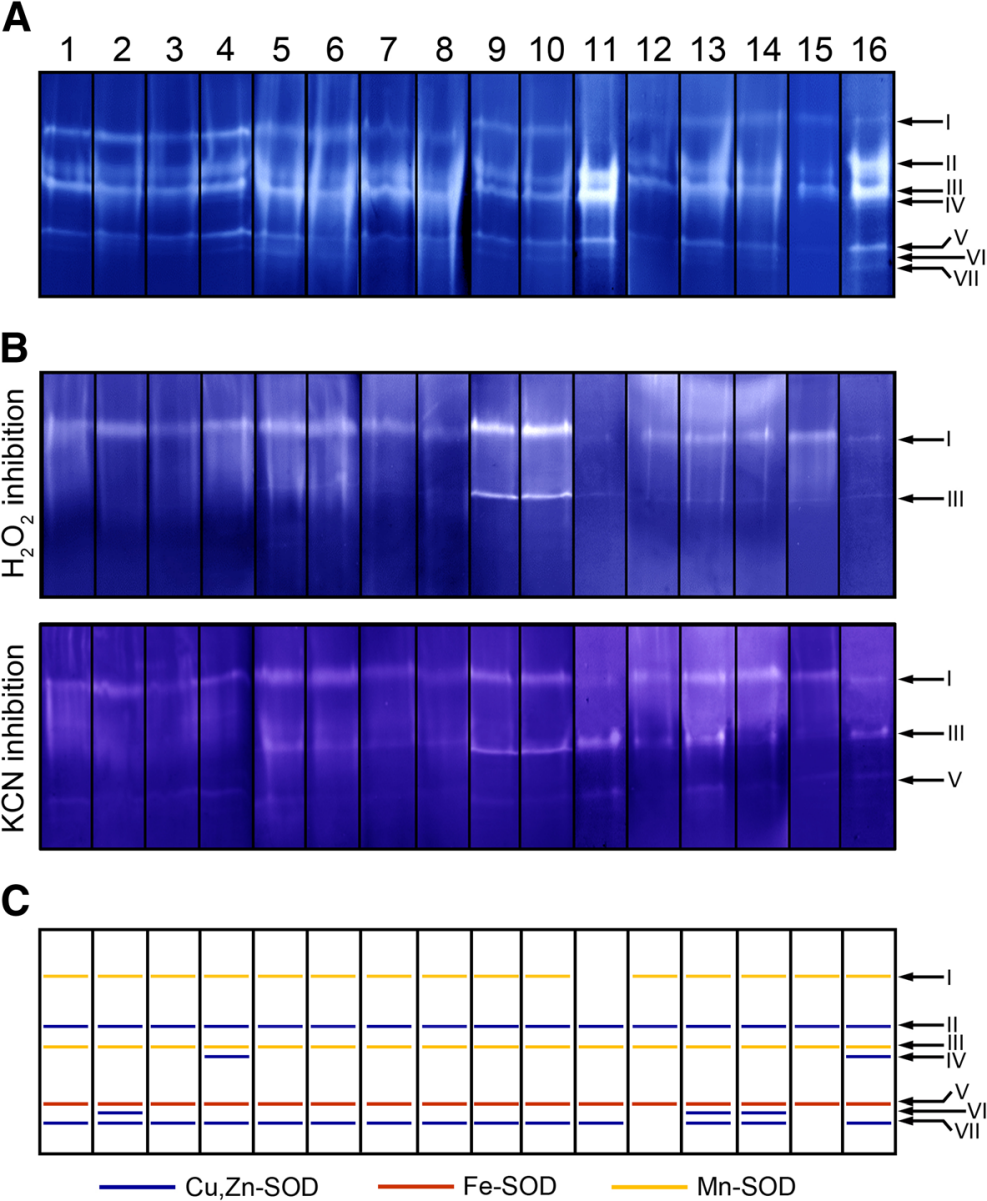
Total SOD activity and inhibition assays of pollen protein extracts from 16 olive cultivars. **a** Total SOD activity of pollen protein extracts from 16 olive cultivars. **b** SOD activity gel after H2O_2_ (upper panel) and KCN (lower panel) inhibition assays, respectively. **c** Type-assignment of the SOD activity bands present in **a** on the basis of MS-based identification data and their inhibition profiles (**b**), numbered from I to VII. Bands marked in yellow are considered Mn-SODs as they are not inhibited neither by H_2_O_2_ nor by KCN. Iron-SODs (red bands) are inhibited by KCN and not by H_2_O_2_, and Cu,Zn-SODs (blue bands) are inhibited by both H_2_O_2_ and KCN. 1, ‘Lechín de Sevilla’; 2, ‘Verdial de Vélez Málaga’; 3, ‘Hojiblanca’; 4, ‘Picudo’; 5, ‘Manzanilla de Sevilla’; 6, ‘Arbequina’; 7, ‘Cornicabra’; 8, ‘Blanqueta’; 9, ‘Loaime’; 10, ‘Lucio’; 11, ‘Bella de España’; 12, ‘Empeltre’; 13, ‘Farga’; 14, ‘Frantoio’; 15, ‘Galega’; 16, ‘Picual’

Distinctive SOD activity band profiles were also observed in native gels depending on the cultivar (Fig. 2a). The presence of Mn-SOD and Cu,Zn-SOD isoenzymes was confirmed by nanoLC-ESI-MS/MS analysis of excised bands from SOD activity gels (cv ‘Picual’). Thus, bands I and III matched with Mn-SOD enzymes, while Cu,Zn-SODs were identified in bands II, IV and VII (see Additional file 1: Table S1). Bands V and VI did not give any positive match in the MS/MS analysis. These data are in good agreement with our results here since transcripts of four distinct cytosolic Cu,Zn-SODs were also identified in the olive pollen transcriptome (Table 1). MS band assignments were also assessed by inhibition assays (Fig. 2b, c). We identified a variable number of Cu/Zn-, Mn-, and Fe-SODs in the olive pollen extracts of all cultivars tested. Interestingly, Mn-SOD activity was found to be significantly higher than either Cu,Zn- or Fe-SOD activities on acrylamide gels. A common pattern of seven bands was observed in most of the cultivars. The upper band (I) corresponded to a Mn-SOD, which was absent in ‘Bella de España’. Bands II and III corresponded to a Cu,Zn- and Mn-SODs, respectively, and were present in all cultivars. Band IV was recognized as a Cu,Zn-SOD and appeared only in ‘Picudo’ and ‘Picual’. Band V was identified as a Fe-SOD in all cultivars. Finally, bands VI and VII also corresponded to Cu,Zn-SODs, which were differentially expressed among cultivars. Accordingly, band VI was displayed in cvs. ‘Verdial de Vélez Málaga’, ‘Farga’, and ‘Frantoio’, whereas band VII was present in all cultivars, except ‘Empeltre’ and ‘Galega’.

**Table 1:** Olive pollen superoxide dismutase (SOD) sequences retrieved from transcriptomic and genomic databases

Gene name	Type	Transcriptome code ^a^	Genome code ^b^	Arabidopsis orthologue (gene name)/Identity (%)^c^	Length (aa)	Mw (Da)/p*I*	Cellular localization
*OeCSD1.1A*	Cu,Zn	po11_olive_011703	OE6A103554T1	At1g08830 (*AtCSD1*)/83	152	15,306.96/5.76 ^d-e^	Cytosol
*OeCSD1.1B*	Cu,Zn	po11_olive_018096	OE6A103554T1	At1g08830 (*AtCSD1*)/78	144	14,521.15/6.09 ^d-e^	Cytosol
*OeCSD1.2*	Cu,Zn	po11_olive_018137	OE6A068806T1	At1g08830 (*AtCSD1*)/83	152	15,232.96/5.93 ^d-e^	Cytosol
*OeCSD1.3*	Cu,Zn	po11_olive_027139	OE6A101614T3	At1g08830 (*AtCSD1*)/82	152	15,200.83/5.76 ^d-e^	Cytosol
*OeCSD2*	Cu,Zn	po11_olive_021051 po11_olive_016542	OE6A004775T3	At2g28190 (*AtCSD2*)/63	230	23,426.38/6.23 ^d^ 23,434.39/6.23 ^e^	Chloroplast
*OeCSD3*	Cu,Zn	po11_olive_018653	OE6A038482T1	At5g18100 (*AtCSD3*)/75	157	15,944.81/6.53 ^d^ 16,045.98/6.53 ^e^	Peroxisome
*OeFSD2*	Fe	po11_olive_023570	OE6A023256T3	At5g51100 (*AtFSD2*)/64	306	35,002.29/5.40 ^d^ 35,051.27/5.24 ^e^	Chloroplast
*OeMSD1*	Mn	po11_olive_008559	OE6A054770T1	At3g10920 (*AtMSD1*)/78	228	25,501.22/6.71 ^d-e^	Mitochondria

^a^ Transcript codes from the olive (cv. ‘Picual’) pollen transcriptome database (http://reprolive.eez.csic.es)

^b^ Transcript codes from the olive (cv. ‘Farga’) genome database (http://denovo.cnag.cat/genomes/olive/)

^c^ Identity (%) between the olive SOD amino acid sequences and the corresponding Arabidopsis orthologues

^d-e^ Molecular weight (Da) and isoelectric point (pI) referred to the transcriptome (d) and genome (e) SOD amino acid sequences, respectively

### Pollen Cu,Zn-SOD enzyme variability in olive cultivars

**Fig. 3:**
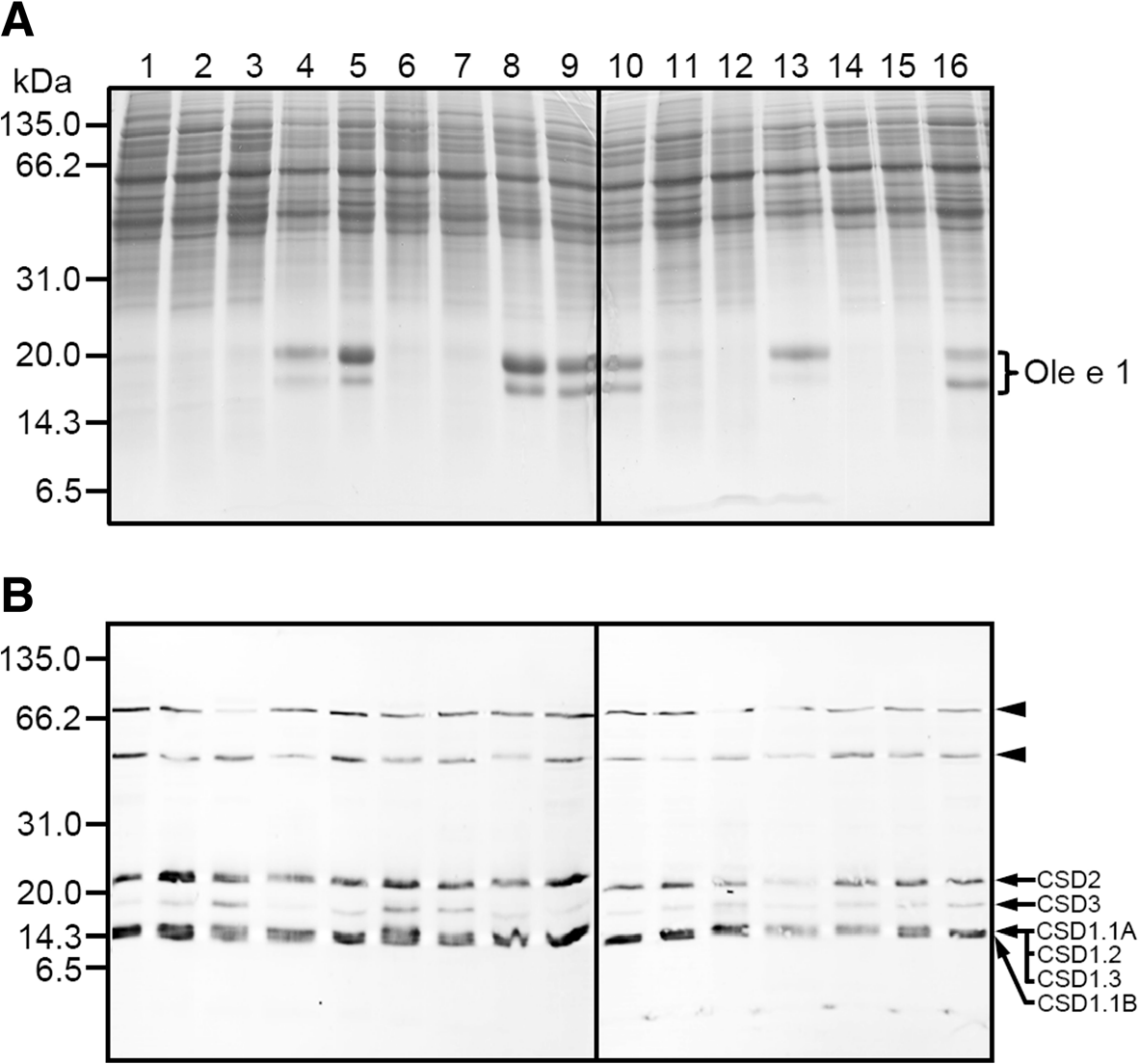
Immunodetection of SOD enzymes in pollen protein extracts from 16 olive cultivars. **a** Total protein profiles of pollen extracts from 16 different olive cultivars after SDS-PAGE. The allergenic protein Ole e 1 is indicated with a bracket. Thirty micrograms of total protein were loaded per lane. Protein markers (kDa) are displayed on the left. **b** Western blot as in A probed with a commercial Ab to a chloroplastidic Cu,Zn-SOD enzyme (anti-CSD2) from *Arabidopsis thaliana*. The antibody was able to recognized a ~ 16 kDa chloroplastic SOD (CSD2) and a 23.4 kDa peroxisomal (CSD3) enzyme. A protein band of 15.2 kDa grouping cytosolic CSD1.1A, CSD1.2 and CSD1.3 forms, and a 14.5 kDa corresponding to the cytosolic CSD1.1B enzyme, respectively, were also visible. Arrows indicate monomeric enzymes, while arrowheads point out putative multimeric forms. 1, ‘Lechín de Sevilla’; 2, ‘Verdial de Vélez Málaga’; 3, ‘Hojiblanca’; 4, ‘Picudo’; 5, ‘Manzanilla de Sevilla’; 6, ‘Arbequina’; 7, ‘Cornicabra’; 8, ‘Blanqueta’; 9, ‘Loaime’; 10, ‘Lucio’; 11, ‘Bella de España’; 12, ‘Empeltre’; 13, ‘Farga’; 14, ‘Frantoio’; 15, ‘Galega’; 16, ‘Picual’

Marked differences in pollen protein profiles were visible in Coomassie-stained 1-D gels among 16 olive cultivars, mainly in relation to a 18–20 kDa band cluster, which correspond to the major olive pollen allergen Ole e 1 (Fig. 3a) according to previous work carried out by the present authors. The corresponding immunoblot probed using an anti-CSD2 antibody revealed up to six different bands in all the extracts, with molecular weights of approximately 72.0, 54.0, 23.5, 16.0, 15.3 and 14.5 kDa (Fig. 3b). On the basis of their theoretical molecular weights, the 23.5 and 16.0 kDa bands likely correspond to chloroplastidic and peroxisomal forms of the Cu,Zn-SOD enzyme (named OeCSD2 and OeCSD3, respectively, after comparison with their *Arabidopsis* orthologues described in the TAIR database: www.arabidopsis.org). Moreover, the 15.3 kDa band might group the cytosolic OeCSD1.1A, OeCSD1.2 and OeCSD1.3 forms, while the 14.5 kDa band can be matched to the OeCSD1.1B enzyme (all these forms also named according to the *Arabidopsis* orthologues present in the TAIR database).

As with 1D, the 2-D gels of whole protein extracts from the olive cultivars ‘Picual’ also exhibited well-represented spots of Ole e 1. After probing the corresponding immunoblot with the Cu,Zn-SOD antibody, different spots with molecular weights of approximately 15, 22, 32, 40 and 60 KDa showed reactivity to the antibody. The overlapping of gel-immunoblotting pictures enabled us to identify the localization of the spots assigned to Cu,Zn-SODs in the 2-D gels of olive pollen extracts after Flamingo staining. The distribution of the immunoreactive spots showed the presence of several isozymes with Ips focused along the whole membrane, which were assigned to putative cytosolic, peroxisomal and plastidial monomeric Cu,Zn-SOD forms, respectively according to the same criteria applying for 1-DE. Several other cross-reactive proteins were not be assigned (see Additional file 2: Figure S1).

**Fig. 4:**
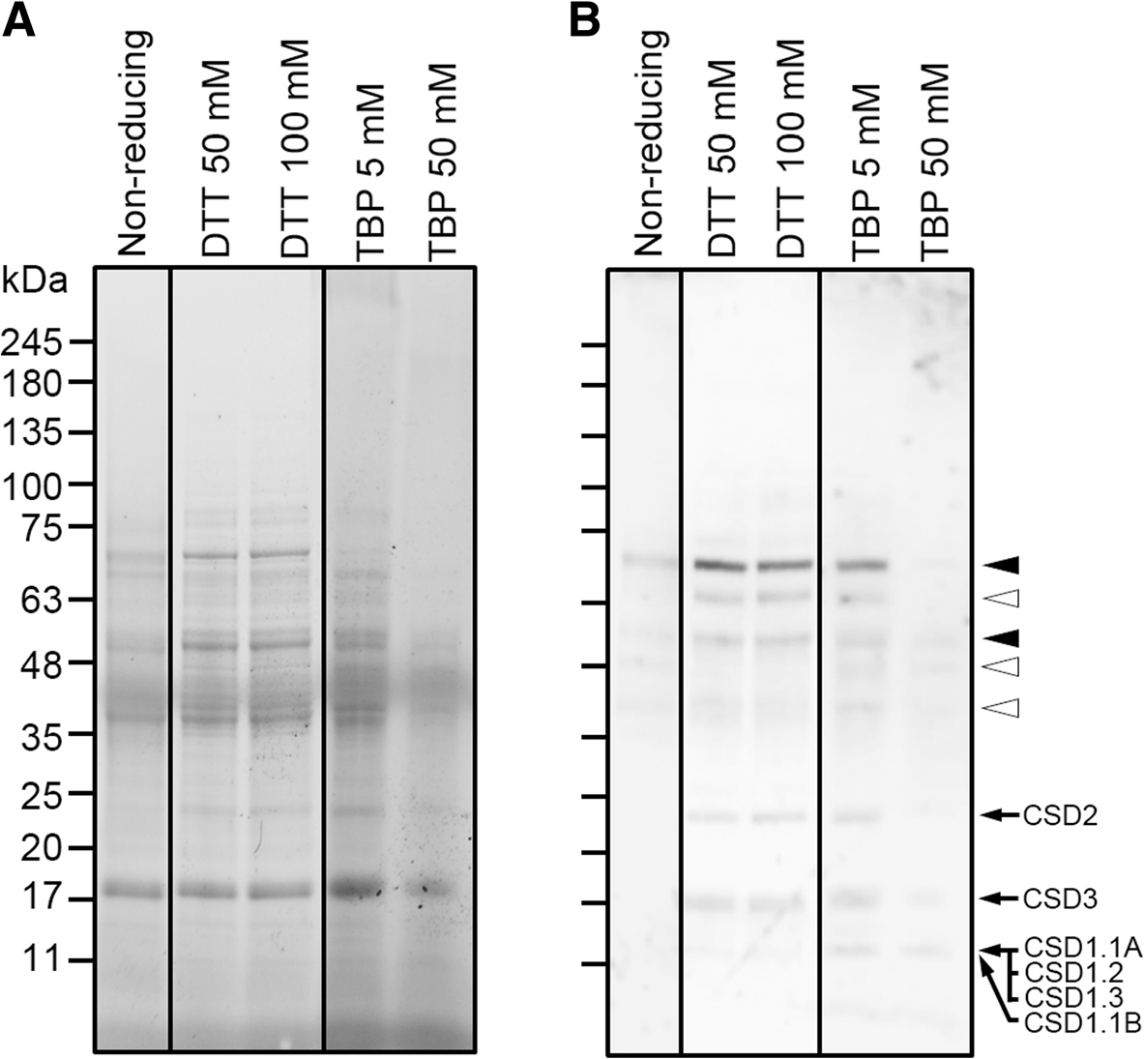
Effect of reducing agents on stability of pollen multimeric SOD enzymes. **a** Total protein 1-D profiles of pollen (cv. ‘Picual’) extracts treated with DTT or tributylphosphine (TBP) at different concentrations prior to SDS-PAGE. Thirty micrograms of total protein were loaded per lane. Protein markers (kDa) are displayed on the left. **b** Western blot as in A probed with a customized anti-olive pollen Cu,Zn-SOD Ab (sequence accession no. EU250769.1). The antibody was able to recognized a ~ 16 kDa chloroplastic SOD (CSD2) and a 23.4 kDa peroxisomal (CSD3) enzyme. A protein band of 15.2 kDa grouping cytosolic CSD1.1A, CSD1.2 and CSD1.3 forms, and a 14.5 kDa corresponding to the cytosolic CSD1.1B enzyme, respectively, were also visible. Arrows indicate monomeric enzymes while black arrowheads point out putative multimeric forms as in Fig. 3. White arrowheads show new putative multimeric SOD forms not detected by the anti-CSD2 antibody

In order to assess whether the observed high molecular weight bands might correspond to multimeric forms of the Cu,Zn-SOD enzymes, we developed new assays by incubating pollen extracts with a panel of reducing and/or denaturing agents, prior to SDS-PAGE and immunoblotting (Fig. 4a). A similar band pattern to that reported with the anti-CSD2 Ab was obtained when probing an anti-olive pollen Cu,Zn-SOD Ab either in 1-D (Fig. 4b) or 2-D (see Additional file 2: Figure S1) blots. In addition, three new high molecular weight polypeptides of ~ 64.0, 48.0 and 42.0 kDa, respectively, were also revealed on immunonoblots when using this anti-olive pollen Cu,Zn SOD Ab (Fig. 4b). The distribution of the bands reacting to the customized antibody was little affected by the treatment of the pollen extracts with chaotropic agents like urea and thiourea (data not shown). The use of DTT favoured the identification of the cytosolic (14.5–15.3 kDa), peroxisomal (16.0 kDa) and chloroplastic (23.5 kDa) monomeric Cu,Zn-SOD forms when compared to the extracts run under non-reducing conditions (Fig. 4b). However, DTT failed to diminish the binding capacity of the anti-olive Cu,Zn-SOD Ab to high molecular weight bands in the range of 42.0 to 72.0 kDa, even at high concentrations. Such bands were only depleted from immunoblots when as much as 50 mM of the reducing agent tributylphosphine (TBP) was used (Fig. 4b).

### Pollen Cu,Zn-SOD enzyme variability in different allergenic plant species

**Fig. 5:**
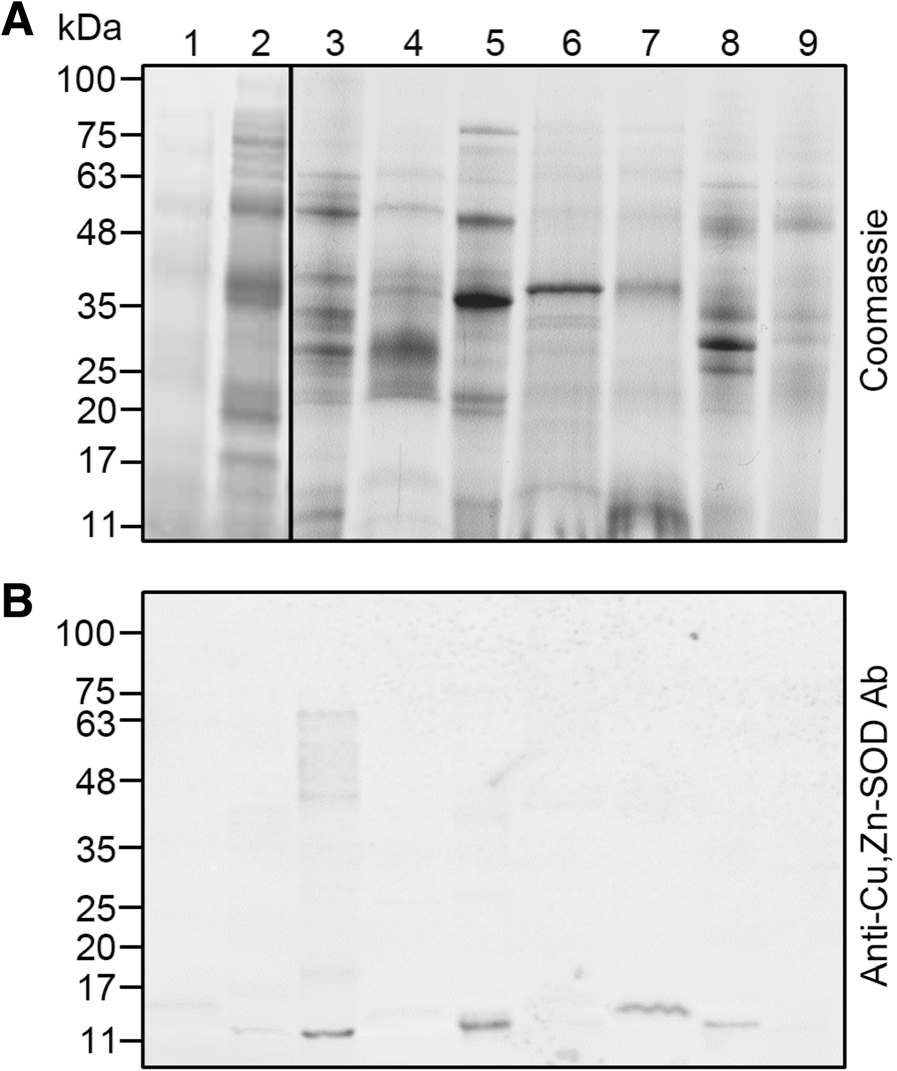
Immunodetection of SOD enzymes in pollen protein extracts of nine allergenic plant species. **a** Total protein profiles of pollen extracts from nine allergenic species after SDS-PAGE. Thirty micrograms of total protein were loaded per lane. Protein markers are displayed on the left. **b** Western blot as in A tested with an anti-olive Cu,Zn-SOD Ab. 1, *Parietaria judaica*; 2, *Salsola kali*; 3, *Phleum pratense*; 4, *Artemisia vulgaris*; 5, *Platanus hybrida*; 6, *Chenopodium album*; 7, *Plantago lanceolata*; 8, *Festuca pratensis*; 9, *Dactylis glomerata*

Given the allergenic nature of Cu,Zn-SOD (also called Ole e 5 allergen), we assessed the presence of Cu,Zn-SODs reactive to the antibody in the pollen of a panel of nine allergenic species. One-dimension gels displayed highly differential protein distribution patterns (Fig. 5). Using the anti-olive Cu,Zn-SOD Ab, a single cross-reactive band was detected in the different species analysed, with sizes ranging from ~ 12.0 to 15.4 kDa. A few additional immunoreactive bands, with molecular weights between ~ 18.1 and 67.4 kDa were also detected in *Phleum pratense*. These molecular weights might indicate the presence of multimeric forms in this species. The *Chenopodium album* and *Dactylis glomerata* pollen extracts showed very faint reactivity to the antibody.

### TEM immunolocalization of Cu,Zn-SOD enzymes in the olive pollen grain

**Fig. 6:**
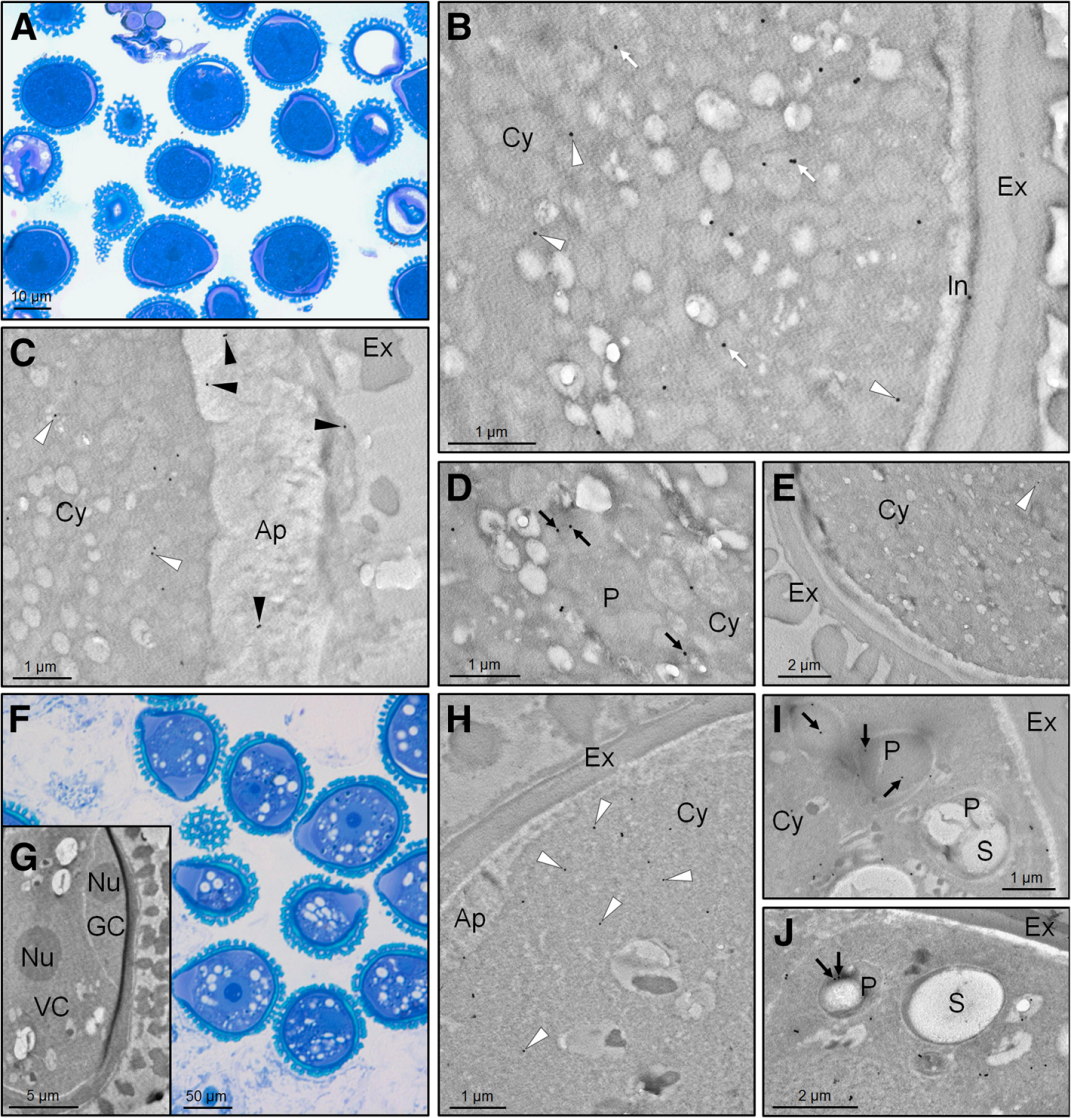
Transmission electron microscopy immunolocalization of SOD enzymes in olive developing pollen grains. **a** Light microscopy methylene blue-stained section of olive mature pollen grains (free pollen grains released from anthers). **b-d** Transmission electron microscopy (TEM) immunolocalization of Cu,Zn-SODs on sections of olive mature pollen grains using an anti-olive Cu,Zn-SOD Ab. **e** Negative control by omitting the primary antibody. **f-g** Light microscopy methylene blue-stained (**f**) and TEM (**g**) sections of olive young pollen grains (prior to anther dehiscence). **h-j** TEM immunolocalization of Cu,Zn-SODs on sections of olive young pollen using the same Ab. Black and white arrowheads indicate exine/aperture and cytosolic locations, respectively. Black arrows show the plastidial SOD, while white arrows point out undetermined-organelle locations of the enzyme. Note that plastids in the young pollen grain may display different degrees of differentiation and presence/absence of starch depending on section orientation. Abbreviations = Ap, aperture; Cy, cytoplasm; Ex, exine; GC, generative cell; I, intine; P, plastid; S, starch; Nu, nucleolus; VC, vegetative cell

Sections of young and mature pollen grains were subjected to immunocytochemical analysis. The mature pollen stage was characterized by the presence of a fully differentiated pollen exine and a vegetative cell that completely engulfs the generative cell (Fig. 6a). At this stage, gold labelling was mainly detected in the cytoplasm of the vegetative cell and the pollen exine (Fig. 6b-c), as well as in poorly differentiated organelles, which may correspond to plastids and peroxisomes (Fig. 6c-d). Negative controls did not show any labelling (Fig. 6e). The young pollen stage was determined by the lateral position of the generative cell (Fig. 6f-g). Labelling was detected in the cytoplasm of the vegetative cell and associated with organelles, some of which were identified as plastids (amyloplasts) due to the presence of starch granules, sometimes polarized in areas of these organelles (Fig. 6h-j).

### *Characterisation of cytosolic* rOeCSD1.1A *and* rOeCSD1.1B *enzymes*

**Fig. 7:**
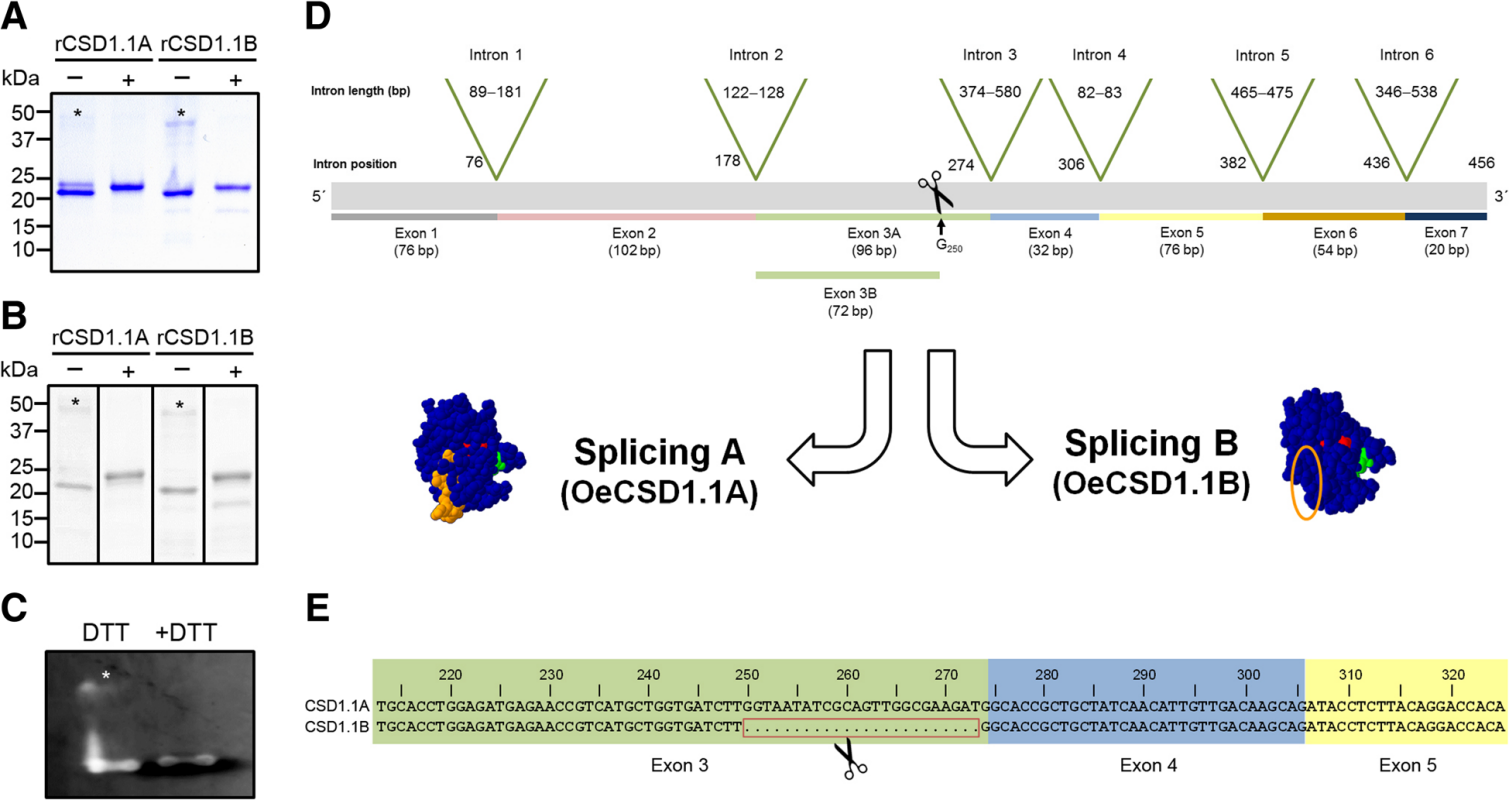
Effect of reducing agents on stability of pollen recombinant SOD enzymes and alternative splicing model. **a** DTT-treated (+) and non-treated (−) of OeCSD1.1A (splicing A form; accession no. EU250770.1) and OeCSD1.1B (splicing B form; EU250769.1) recombinant SODs were electrophoresed and stained with CBB. Ten micrograms of protein were loaded per lane. Protein markers are displayed on the left. Asterisks indicate dimeric SOD forms. **b** Western blot as in A tested with an anti-olive Cu,Zn-SOD Ab. Asterisks show SOD dimers. **c** *In-gel* SOD activity of DTT-treated (+DTT) and non-treated (−DTT) rOeCSD1.1A proteins. Asterisk indicates an rOeCSD1.1A dimer. **d** Schematic representation (exons and introns) of olive pollen *OeCSD1.1A* and *OeCSD1.1B* genes. Green arrowheads point out the position and length of the introns in the cDNA. A total of six introns were predicted by using the bioinformatics tool provided by Cruz et al. (2016). The position and length of the seven exons, as well as the alternative splicing of exon 3, are also indicated. The 3D models of both the OeCSD1.1A and OeCSD1.1B forms of the enzyme are shown. The missing fragment in the OeCSD1.1B form is indicated with an orange circle. **e** Detail of the nucleotide sequence at the alternative splicing zone in the OeCSD1.1A and OeCSD1.1B transcripts

The identity of the full length (OeCSD1.1A) and short (OeCSD1.1A) forms of a recombinant cytosolic Cu,Zn-SOD was confirmed by MALDI-TOF/TOF (see Additional file 3: Table S2). The molecular weights of these recombinant proteins, which were expressed as His-tag fusion proteins, were determined by SDS-PAGE to be approximately 22.1 and 21.3 kDa, respectively (Fig. 7a). In turn, theoretical molecular weights calculated from their amino acid sequences are 15,306.90 and 14,521.15 Da, respectively. Differences between theoretical and empirical Mw values are likely due to the presence of the His-tag in the recombinant forms. Additional rOeCSD1.1A and OeCSD1.1B bands of ~ 44.5 and ~ 42.8 kDa, respectively, were also observed under non reducing conditions (Fig. 7a, asterisks). These protein bands likely correspond to dimeric forms since they were removed from gels after DTT treatment. The anti-Cu/Zn-SOD antibody recognized the monomeric and dimeric forms (Fig. 7b), which were both active under non-reducing conditions (Fig. 7c). However, after reduction, only the monomeric enzyme conserved its activity. Parallel sets of native acrylamide gels including both recombinant proteins and pollen total protein extracts were subjected to SOD activity and native immunoblotting assays with the customized anti-olive Cu,Zn-SOD Ab (see Additional file 4: Figure S2). Both recombinant proteins were active, although the shorter enzyme showed lower activity levels. Moreover, a good correspondence between the activity and Ab-reacting bands in terms of number and gel position was observed.

## Discussion

### SOD activity in pollen is higher than in vegetative tissues and is not correlated to viability

Superoxide dismutase activity in pollen was highly variable and dependent on the cultivar assayed, with values ranging from 5 to 25 U/mg protein. Pollen samples in our study were collected at the same developmental stage and on the same day, and trees of all cultivars analysed were located close to each other. Therefore, we should not expect a major influence of environmental factors over SOD activity rates, and differences among cultivars should be rather due to their distinct genetic background. Reactive oxygen species homeostasis is critical for proper pollen functioning. Thus, ROS, and particularly H_2_O_2,_ participate as signals in diverse pollen-pistil interaction processes [7] such as self-incompatibility [7, 44, 45], as well as in events driving the polarization of the pollen tube during its apical growth [45–47]. Hence, the high rates of SOD activity observed might contribute to balance the extremely high metabolic rates of the pollen grain upon hydration and germination. Moreover, previous studies showed a direct correlation between an increase in Cu,Zn-SOD activity and tolerance to dryness in several species [48, 49]. This fact suggests that high levels of SOD activity might also protect the highly dehydrated mature pollen during its journey from the dehiscent anther to the receptive stigma. The lack of correlation between SOD activity and pollen viability in these cultivars may have different explanations. Firstly, the FCR-based viability test is based on the presence of active esterases rather than SODs [50]. Secondly, although SODs may accumulate prior to the mature stage, their function is likely to be required at higher rates later, upon pollen hydration and during germination and pollen tube growth.

### The olive pollen grain contains a full set of Cu,Zn-, Mn- and Fe-SOD isoenzymes

The presence of up to four cytosolic Cu,Zn-SOD forms in the olive pollen (cv. ‘Picual’) was previously described after isoelectric focusing experiments [41]. After comparison with the transcriptomic data showed here, the ~ 15.3 kDa band observed on immunoblots, and initially assigned as a single polypeptide, might indeed comprise three different cytosolic Cu,Zn-SOD forms, namely CSD1.1A, CSD1.2 and CSD1.3. These SOD forms showed very similar theoretical molecular weights ranging from 15.2 to 15.3 kDa, so that they could not be discriminated after 1-D electrophoresis (Table 1). However, 2-D electrophoretic separations allowed us to distinguish them on the basis of their distinct isoelectric points (see Additional file 2: Figure S1). An eight-aa shorter form of ~ 14.5 kDa was also identified and is discussed below. The four amino acid sequences showed 100% identity in cultivars ‘Picual’ and ‘Farga’, suggesting that these proteins might be highly conserved in the olive germplasm.

Two additional Fe-SOD and Mn-SOD isoenzymes were identified in the pollen grain of this species. The presence of the corresponding transcripts was also supported by transcriptomic data (Table 1). Previous work demonstrated the expression of a Mn-SOD gene at very low levels in the pollen grain of maize [50]. However, to our knowledge, this is the first time that Mn-SOD and Fe-SODs have been clearly demonstrated to be present at protein level in the male gametophyte. Iron-SODs are typically associated with chloroplasts [21], while Mn-SOD isoenzymes are present in mitochondria and peroxisomes [26, 27]. Interestingly, the cytoplasm of the vegetative cell, as well as the pollen tube in the region behind the apex, contains abundant mitochondria and plastids, both of which are involved in energy supply during pollen maturation and pollen tube growth [51, 52]. This fact suggests that pollen Mn- and Fe-SOD enzymes might be involved in ROS homeostasis in these organelles during development.

### Plastidial and peroxisomal Cu,Zn-SOD enzymes are present in the pollen grain

Based on biochemical and transcriptomic data, we suggest that the 16.0 and 23.5 kDa bands observed on immunoblots likely correspond to monomeric forms of peroxisomal and chloroplastic Cu,Zn-SOD proteins, respectively. Frequently, those customized antibodies originally raised against cytosolic Cu,Zn-SODs may also recognize their chloroplastidic [53] and peroxisomal [39] counterparts due to high homology between both sequences. Some commercially available antibodies to cytosolic Cu,Zn-SODs have also been reported to cross-react to some degree with their chloroplastidic and peroxisomal equivalents, as it is clearly corroborated in the present work. The presence of post-translational modifications might explain the high number of spots detected on 2-D immunoblots (see Additional file 2: Figure S1), although there is no direct evidence supporting this hypothesis [54–56].

Chloroplastidic Cu,Zn-SODs were reported to be present in the thylakoid membranes of the vegetative tissues at the stromal face close to the PSI [57]. A pioneer study carried out on SODs in mature olive pollen [41] failed to report the presence of chloroplastidic Cu,Zn-SOD forms. This might be explained by the fact that olive mature pollen organelles are often undifferentiated with, in many cases, plastids, mitochondria and peroxisomes being indistinguishable unless specific preparation methods or markers are used [39]. The presence of amyloplasts, a type of plastid responsible for the synthesis and storage of starch, in mature pollen grains has been described previously by [58]. It has been suggested that plastidial glycolysis together with mitochondrial respiration, fermentation and cytosolic glycolysis are some of the ways in which energy for the metabolism during pollen maturation and pollen tube growth is obtained [52]. Here, we reported the localization of a Cu,Zn-SOD enzyme in subcellular structures that are compatible with plastids in mature pollen grains. At younger stages, the signal was clearly attributed to amyloplasts, which were easily recognizable by their starch content. Two partial nucleotide sequences annotated as chloroplastidic Cu,Zn-SODs were identified in the olive (cv. ‘Picual’) pollen transcriptome (Table 1). These two partial amino acid sequences overlap to generate a complete sequence that displayed 99.13% identity with a chloroplastidic Cu,Zn-SOD present in the olive (cv. ‘Farga’) genome.

**Fig. 8:**
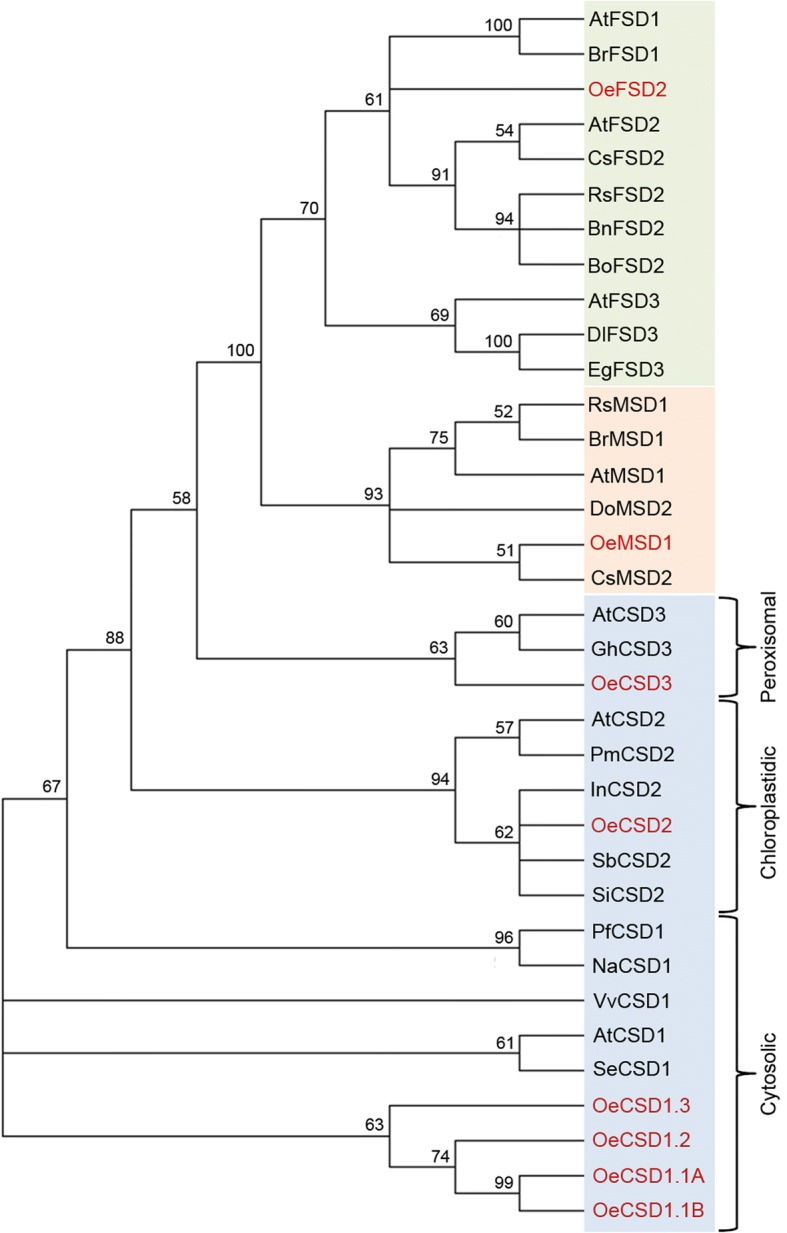
Molecular phylogenetic analysis of superoxide dismutase nucleotide sequences by Maximum Likelihood (ML) method based on the General Time Reversible (GTR) model and conducted in MEGA7 [79]. The bootstrap consensus tree was inferred from 100 replicates. The percentage of replicate trees in which the associated taxa clustered together in the bootstrap test (100 replicates) are shown next to the branches. Initial tree(s) for the heuristic search were obtained automatically by applying Neighbor-Join and BioNJ algorithms to a matrix of pairwise distances estimated using the Maximum Composite Likelihood (MCL) approach, and then selecting the topology with superior log likelihood value. A discrete Gamma distribution was used to model evolutionary rate differences among sites [4 categories (+G, parameter = 1,9145)]. The analysis involved 35 nucleotide sequences [AtCSD1 (NCBI accession no. AY091168); AtCSD2 (AY133756); AtCSD3 (BT003689), AtFSD1 (AF324711), AtFSD2 (BT005116), AtFSD3 (AY091225), AtMSD1 (NM111929), BnFSD2 (XM_013827408.1), BoFSD2 (XM_013774046.1), BrFSD1 (HQ258931.1), BrMSD1 (XM_009124281.2), CsFSD2 (XM_010444287.2), CsMSD2 (KP189420.1), DlFSD3 (KT894098.1), DoMSD3 (JQ797737.1), EgFSD3 (KU904638.1), GhCSD3 (EU597270.1), InCSD2 (XM_019331435.1), NaCSD1 (JN869247.1), OeCSD1.1A (transcript code po11_olive_011703), OeCSD1.1B (po11_olive_018096), OeCSD1.2 (po11_olive_018137), OeCSD1.3 (po11_olive_027139), OeCSD2 (po11_olive_021051 + po11_olive_016542), OeCSD3 (po11_olive_018653), OeFSD2 (po11_olive_023570), OeMSD1 (po11_olive_008559), PfCSD1 (GU731670.1), PmCSD2 (XM_008228408.1), RsFSD2 (XM_018621411.1), RsMSD1 (XM_018578014.1), SbCSD2 (HQ395747.1), SeCSD1 (JQ074238.2), SiCSD2 (XM_011090495.1), VvCSD1 (JQ692111.2)]. Olive pollen SOD sequences are in red. Blue, orange and green squares represent Cu,Zn-, Mn- and Fe-SODs, respectively. Among the Cu,Zn-SODs, the three subclades corresponding to the cytosolic, chloroplastidic and peroxisomal forms are indicated with brackets. Abbreviations: At, *Arabidopsis thaliana*; Bn, *Brassica napus*; Bo, *Brassica oleracea*; Br, *Brassica rapa*; Cs, *Camelina sativa*; Dl, *Dimocarpus longan*; Do, Diospyros oleifera; Eg, *Eucalyptus grandis*; Gh, *Gossypium hirsutum*; In, *Ipomoea nil*; Na, Neosinocalamus affinis; Oe, *Olea europaea*; Pf, *Pleioblastus fortunei*; Pm, *Prunus mume*; Rs, *Raphanus sativus*; Sb, Scutellaria baicalensis; Se, *Salicornia europaea*; Si, *Sesamum indicum*; Vv, *Vitis vinifera*

The cytoplasm of both the vegetative cell and the pollen tube also contain peroxisomes [59]. A recent work unequivocally demonstrated the presence of a Cu,Zn-SOD enzyme in the olive pollen peroxisomes by co-localization experiments at TEM using a customized anti-Cu,Zn-SOD Ab and an antibody reactive to catalase, which is a peroxisomal marker enzyme [39]. In the present study, the existence of specific peroxisomal Cu,Zn-SOD transcripts was also supported by transcriptomic data (Table 1). Thus a complete amino acid sequence showed 99.35% identity with a sequence from the olive genome (Table 1). The molecular phylogenetic analysis of olive pollen SODs grouped separately Cu,Zn-, Fe and Mn-SODs in three clades (Fig. 8). Within the Cu,Zn-SOD clade, cytosolic, chloroplastidic and peroxisomal Cu,Zn-SODs were placed in separate branches, each with other similar plant Cu,Zn-SODs (Fig. 8).

### Pollen Cu,Zn-SODs might form multimeric enzyme complexes

The high molecular weight SOD bands observed on immunoblots might correspond to multimeric forms of these enzymes. Indeed, the presence of high molecular weight SOD isoenzymes resisting treatments with reducing agents like DTT or β-mercaptoethanol has been shown repeatedly, although it has been frequently unnoticed [55]. Other authors have described Cu,Zn-SOD enzymes as some of the most stable globular protein families studied. They maintain a stable native dimeric structure at high temperatures even under reducing conditions, due to hydrophobic interfaces, the packing of the stranded β-barrel scaffold, the presence of an intra-chain disulphide bridge and the stabilizing effect of metal cofactors [60].

Alternatively, plant proteins other than SODs may also catalyse the disproportionation of O_2_^**·-**^ radicals. This is the case of germin, a manganese containing protein, structurally equivalent to 7S seed storage proteins (vicilins) with bifunctional (oxalate oxidase and superoxide dismutase) characteristics [61, 62]. Germins are prone to aggregation forming pentamers to, more frequently, hexamers of high molecular weight [62, 63], and are unusually resistant to proteases, detergents and heat [63–65]. At least 22 transcripts were identified in olive pollen (http://reprolive.eez.csic.es/) with relevant homology to this protein (data not shown). Validation of both expression and activity of this enzyme in olive and other species is yet to be carried out.

### Ole e 5-like proteins are ubiquitous in the pollen grain of allergenic species

Copper,Zinc-SODs and Mn-SODs have been reported to be allergenic proteins in a variety of sources including insects (cockroaches, *Drosophila*), fungi (*Alternaria*, *Aspergillus*, *Penicillium*, *Cochliobolus*, *Malassezia*), grasses (*Phleum pratense*), tomato (*Lycopersicon esculentum*), olive (*Olea europaea*), rubber tree (*Hevea brasilensis*) and *Pistacia sps.* (www.allergome.org/index.php and literature therein). In this study, we analysed the presence of Ole e 5-like (Cu,Zn-SOD) allergens in a relatively large number of species documented in terms of their ability to produce allergy-eliciting pollen. We concluded that all of them carried Ole e 5-like forms, which are cross-recognized by the anti-olive Cu,Zn-SOD Ab. Ole e 5 allergen is considered to be a minor allergen of the olive pollen, which, despite its low prevalence, causes allergic symptoms and IgE and/or skin prick test (SPT) reactivity in about 35% of the population analysed that is allergic to this pollen [42]. Cross-reactivity of the allergen among species has not yet been extensively analysed [66]. However, on the basis of the results obtained in this study, Cu,Zn-SODs and other SOD enzymes are probably involved in the complex allergograms of the species we have analysed. To support this hypothesis, we took into account the similar molecular weights of the cross-reacting bands, the enzyme’s high conservation levels in many different sources and its rapid and easy release from pollen grains among other factors. All this needs to be further analysed using specific techniques such as SPTs, ELISA and immunoCAP techniques.

### The olive pollen contains a novel shorter Cu,Zn-SOD form with maintained functionality

One of the cytosolic Cu,Zn-SOD sequences (OeCSD1.1B) identified in the olive pollen transcriptome included an important 24 nt gap. Multiple sequences of Cu,Zn-SOD harbouring this gap have been also identified in the genome of *O. europaea* cv. ‘Farga’, recently sequenced and annotated [67], as well as in the transcriptomes generated from leaf, root and fruit tissues [68]. We suggest that this shorter Cu,Zn-SOD form could be the result of an alternative splicing event (Fig. 7d-e). Following further in silico structural analysis, we were able to predict that the resulting gene products may be both active enzymes. Thus, the functionality of the CSD1.1B sequence assembled after NGS and that cloned by the authors, was unlikely to be greatly affected, as neither the reading frame nor the presence of key amino acids was disturbed. Accordingly, the heterologous expression of the OeCSD1.1A and OeCSD1.1B enzymes resulted, in both cases, in proper folding to at least a certain degree, though in a small proportion of both proteins, as demonstrated by the detection of SOD activity in both expressed forms. Noticeably, the full lenght SOD enzyme showed higher levels of activity than the shorter one. Therefore, it is possible that the removal of the 8-AA fragment not only slightly affected the molecular weight and isoelectric point of the protein but it might also lead to subtle changes in its activity and/or kinetics. We also observed the spontaneous appearance of low amounts of dimeric forms of the recombinant proteins, very likely through the formation of intermolecular disulphide bridges. Interestingly, both the monomeric and dimeric enzymes were shown to be active on native gels.

On the other hand, the stability of the recombinant enzymes was very low. Thus, no reproducible results were obtained when spectrophotometric assays of SOD activity were carried out (data not shown). Characteristics such as instability and loss of activity have been widely described for monomeric forms [69–72]. Other factors including the lack of adequate concentrations of the ions needed for proper folding might have also led to the loss of protein stability. Thus, the Zn atom has been reported to directly influence the Cu site by modifying its reduction potential and geometry [29]. The negative charge at physiological pH has also been linked to Cu,Zn-SOD stability [73]. Further improvements in the production of these proteins should enable the differential properties of these enzyme forms to be tested.

The absence of the described peptide in the shorter form of the protein might also affect its antigenic and allergenic properties, as the short missing sequence has been predicted to take part of different T- and B- epitopes [43]. This should be further investigated as well, due to the relevant properties of the protein as allergen (Ole e 5).

## Conclusions

The olive pollen grain contains a full set of SOD enzymes, including cytosolic, plastidial and peroxisomal Cu,Zn-SOD forms, as well as Mn- and Fe-SOD isoenzymes. In addition, we identified a less abundant 8 aa-shorter Cu,Zn-SOD form (likely the product of an alternative splicing event), which is fully functional. Interestingly, the level of SOD activity is cultivar-dependent, probably as a consequence of the intrinsic genetic variability of the olive tree germplasm. The presence of this versatile enzymatic battery and the high levels of SOD activity detected in the pollen grain, together with the plethora of other antioxidant enzymes and factors responsable for maintaining the structure of macromolecules and membranes, might confer it resistance to desiccation and other adverse environmental factors during its journey from the dehiscent anther to the receptive stigma, and might also respond to the necessity of finely regulating the ROS homeostasis during the period of highly active metabolism that follows its germination.

## Methods

### Plant material

Mature pollen samples from 16 different olive (*Olea europaea* L.) cultivars were obtained from trees of the germplasm collection at C.I.F.A. Venta del Llano (Mengíbar, Jaén, Spain). Pollen samples were collected in paper bags by vigorously shaking the olive inflorescences, sieved through 150 and 50 μm mesh nylon filters to remove debris, and then stored at − 80 °C.

Mature pollen samples from *Parietaria judaica, Salsola kali, Phleum pratense, Artemisia vulgaris, Platanus hybrida, Chenopodium album, Plantago lanceolata, Festuca pratensis* and *Dactylis glomerata* were provided by Inmunal SAU (Madrid, Spain).

### Pollen viability assay

Pollen viability was assayed using fluorescein diacetate (Sigma, USA) as described by [49]. Observations were carried out under an Axioplan fluorescence microscope (Zeiss, Germany) equipped with a ProGres C3 CCD camera, using the ProGres CapturePro 2.6 software (Jenoptik AG, Germany). At least 1000 pollen grains per cultivar, corresponding to three independent biological replicas, were counted.

### Protein extraction and quantification

Pollen protein extracts from 16 olive varieties were prepared by stirring 0.1 g of material in 1.5 ml of extracting buffer [50 mM phosphate buffer (pH 7.8) and 1 mM phenylmethylsulfonyl fluoride (PMSF)] for 4 h at 4 °C. Samples were centrifuged at 12,000 g for 20 min at 4 °C, and the supernatants were sieved through a 0.22 μm filter (MillexGP, Millipore, USA) and stored at − 20 °C until use. For 2-DE, protein extracts from ‘Picual’ and ‘Arbequina’ pollen samples were prepared as described before [74].

Pollen protein extracts from allergenic plant species were obtained by stirring 0.1 g of material in 2.5 ml of extracting buffer [40 mM Tris-HCl (pH 7.0), 2% (*v*/v) Triton X-100, 60 mM dithiothreitol (DTT) and 10 μl of protease inhibitor cocktail (Sigma)] for 2 h at 4 °C. Protein extracts were desalted using PD-10 columns (GE Healthcare Biosciences AB, Sweden) and concentrated with 20% (*w*/*v*) trichloroacetic acid (TCA) in chilled acetone overnight. Samples were centrifuged at 10,000 g for 30 min at 4 °C, and pellets were resuspended in 40 mM Tris-HCl (pH 8.8).

Assessment of the effects of reducing and denaturing agents on multimeric SODs was performed by incubating pollen extracts with DTT (50–200 mM) or tributylphosphine (TBP) (5–50 mM), and urea (3.5 M) and/or thiourea (1 M), respectively, prior to electrophoresis. All protein extracts were quantified using the 2D Quant Kit (Amersham Biosciences, UK) according to the manufacturer’s instructions.

### SOD activity

Total SOD activity of protein extracts was assayed in vitro according to the method of [14]. One unit (U) of SOD activity was defined as the amount of enzyme that inhibits the rate of reduction of cytochrome *c* by 50% in a coupled system, using xanthine and xanthine oxidase, at pH 7.8 and 25 °C. All activity assays were performed in triplicate.

Pollen SOD isoenzymes were separated by native PAGE on 15% polyacrylamide gels using a Hoefer electrophoresis unit SE 600 series (Amersham Pharmacia Biotech, UK), and their activity was visualized as described by [75]. Approximately 375 μg of total protein was loaded per lane. Parallel native PAGE gels were also incubated in the reaction buffer [5 mM H_2_O_2_ or 2 mM KCN] for 20 min in the dark before SOD activity detection as above. These experiments were done in duplicate.

### Production of recombinant SOD proteins

Two olive pollen sequences (accession no. EU250770.1 and EU250769.1), representative of the full length and 8 aa-shorter OeCSD1.1 forms, respectively, were used to generate the recombinant proteins (see Additional file 5: Figure S3). Both sequences were cloned into the pKB6 vector (Rekom Biotech SL, Spain) to generate the constructs SODc-pKB6 and SODd-pKB6 (see Additional file 6: Figure S4). Both proteins were expressed in *E. coli* (Rosetta BL21 pLysS strain) grown at 37 °C, after induction with 1 mM isopropil-β-D-1-thiogalactopyranoside (IPTG) for 2 h. Recombinant SODs were captured using a Ni Sepharose 6 Fast Flow resin (GE Healthcare Biosciences) by affinity chromatography to the His tag fused to the N-terminus of each protein. Recovered protein solutions were further refined and concentrated by anion exchange chromatography (Q Sepharose™ Fast Flow, GE Healthcare Biosciences) and microfiltration using an Acrodisc® Syringe Filter 0,2 μm HT Tuffryn® low protein binding membrane (Pall Corporation, USA). Recombinant proteins were quantified at 280 nm, using the theoretical extinction coefficient [76], and stored at − 80 °C in 20 mM sodium phosphate (pH 8.0) and 1 M NaCl until use.

### SOD identification by mass spectrometry

The identity of recombinant SOD enzymes was confirmed by MALDI-TOF/TOF MS analysis at the Proteomic Facilities of the López-Neyra Institute of Parasitology and Biomedicine (CSIC, Granada, Spain). Briefly, proteins were reduced, alkylated and enzymatically digested following standard procedures. Tryptic digests (1 μl) were mixed with a matrix solution (1 μl) of α-cyano-4-hydroxycinnamic acid (CHCA), and applied to an AnchorChip target plate (Bruker-Daltonics, Billerica, Massachusetts, USA). MS spectra were obtained using an Ultraflex Xtreme MALDI-TOF/TOF mass spectrometer (Bruker-Daltonics) using Flex Control v3.4 software (Bruker-Daltonics), and processed by ProteinScape v3.1.3 software (Bruker-Daltonics). The spectrometer was externally calibrated using the Peptide Calibration Standard mixture (Bruker). The peptide mass fingerprint (PMF) search was performed against the ReprOlive database (21,607 sequences, 3,557,391 residues; [68] using MASCOT 2.4.0 software (Matrix Science, UK) integrated with ProteinScape software (Bruker-Daltonics, Germany). An error of less than 50 ppm on the parent ion mass was tolerated. One missed cleavage per peptide was allowed, and carbamidomethylation for Cys as fixed variable and oxidation for Met as variable modification were set. There were no constrains with respect to protein Mw and p*I*. The signal to noise threshold ratio was set to 3. The significance threshold was set at bp 0.05.

NanoLC-MS/MS analyses of excised bands from SOD activity gels were performed on a nanoACQUITY Ultra Performance LC® System (UPLC®) coupled to a quadrupole time-of-flight mass spectrometer (maXis; Bruker Daltonics) equipped with a nano-electrospray source. Mass data were searched using a local Mascot server (v2.4.0; MatrixScience) against Viridiplantae database (Taxonomy ID 33090). Searches were performed without any Mw or p*I* restrictions and carbamidomethylation for Cys and oxidation for Met were set as variable modifications. Mass tolerances on precursor and fragment ions of 20 ppm and 0.07 Da were set, respectively. Mascot (.dat) results were filtered to evaluate the false discovery rate [77]. Protein identification was confirmed when at least two peptides with high-quality MS/MS spectra (less than 10 points below Mascot’s threshold score of identity at a 95% confidence level) were identified. This threshold led to protein identification with a false discovery rate of less than 1.5%.

### Antibody generation

The polyclonal antibody against the olive pollen Cu,Zn-SOD was generated in two different rabbits after immunization with the whole recombinant OeCSD1.1.B protein (accession no. EU250769.1) by Davids Biotechnologie (Germany). Specificity of this antibody was further established by comparing its reactivity with that of an anti-CSD2 (*Arabidopsis thaliana* chloroplastidic Cu,Zn-SOD; At2g28190) antibody (Agrisera, Sweden).

### Western blotting

Proteins were separated and electroblotted using the stain-free technology (Bio-Rad) according to the manufacturer’s instructions. After blotting, polyvinylidene fluoride (PVDF) membranes were blocked with 10% (*w*/*v*) defatted milk for 1 h, and incubated with the anti-Cu/Zn SOD Ab (diluted 1/5000) for 12 h at 4 °C. Immunodetection was performed using an anti-rabbit IgG Ab conjugated with Alexa Fluor 532 (Molecular Probes, UK) (diluted 1/10,000) for 1 h. Protein bands were visualized in a Pharos FX system (Bio-Rad, USA) and molecular weights were calculated using the Quantity One Software v.4.6.2 (Bio-Rad). All experiments were performed in duplicate.

### Two-dimensional electrophoresis

Rehydration of 11 cm IPG strips (pH 3–10 NL, BioRad), including the sample (200 μg of total protein) in the rehydration buffer, was performed overnight. Isoelectric focusing (IEF) was carried out at 20 °C in an Ettan IPGphor IEF apparatus (Amersham Biosciences) as follows: 300 V for 1 min, 1000 V for 10 min, 8000 V for 90 min and finally a total of 30 kVh. The reduction and alkylation steps were performed as previously described [78]. The second dimension was carried out in a Criterion™ Cell (Bio-Rad) and gels were stained with Coomassie brilliant blue (CBB). Immunoblotting was carried out in parallel as described above.

### Preparation of samples for TEM immunocytochemistry

Mature pollen grains from cv. ‘Picual’ were fixed with 4% (w/v) paraformaldehyde and 0.2% (*v*/v) glutaraldehyde in 0.1 M cacodylate buffer (pH 7.2) for 24 h at 4 °C. Samples were dehydrated throughout an ethanol series, and embedded in Unicryl resin (BBInternational, UK). Ultra-thin (70 nm) sections were obtained using a Reichert-Jung Ultracut E microtome (Leica Microsystems, Germany) and mounted on Formvar-coated 200 mesh nickel grids.

For immunolocalization, sections were blocked with 1% (w/v) bovine serum albumin (BSA) in PBS solution for 1 h, followed by incubation with the anti-Cu/Zn SOD antibody (diluted 1/200) at 4 °C overnight. After three washes of 5 min each with PBS, sections were incubated with an anti-rabbit IgG-30 nm gold-conjugated Ab (BBInternational) (diluted 1/50 in PBS containing 2% BSA). Finally, sections were contrasted with 5% (v/v) uranyl acetate for 20 min and examined under a JEM-1011 electron microscope (JEOL, Japan).

### Bioinformatic analyses

The alignment of nucleotide sequences was performed by using the Clustal W software (http://www.ebi.ac.uk/Tools/clustalw/). A phylogenetic tree of all SOD amino acid sequences from olive pollen and *Arabidopsis thaliana* was constructed in MEGA 7.0 by the maximum likelihood (ML) method [79]. The splicing A and B forms of OeCSD1.1 were subjected to 3D reconstruction (http://swissmodel.expasy.org/workspace/) [80] by using the 2q21 template (annotated as a dimer) available as PDB by means of the DeepView v3.7 software. Search of olive SOD genomic sequences was performed using the BLAST tool as described by [67].

### Statistical analyses

The Kolmogorov-Smirnov test was used to test the normality of SOD activity and pollen viability data. An ANOVA analysis was performed to assess differences in both SOD activities and pollen viability among cultivars. Pollen SOD activity levels and viability were also compared using ‘Picual’ as the reference cultivar. Statistical comparisons were carried out using the Student’ s *t* test at a significance level of *p* < 0.01. The Levene’s test was applied to assessed the equality of variances. The Pearson test was carried out in order to determine whether SOD activity and pollen viability variables were correlated. All analyses were performed using SPSS v.23 software (IBM, USA).

## Additional files

Additional file 1: **Table S1.** Identification of protein bands (I-VII) from SOD activity native gels (cv. ‘Picual’) by nano-LC-ESI-MS/MS analysis. (PDF 238 kb)

Additional file 2: **Figure S1.** Immunodetection of Cu,Zn-SOD enzymes in olive (cv. ‘Picual’) pollen protein extracts by 2-D Western blotting. Coomassie brilliant blue (CBB)-stained gel (left) and immunoblot probed with an anti-olive Cu,Zn-SOD antibody (right). Two hundred micrograms of total protein were loaded on the IEF strips. Protein markers are shown on the left. Circles, triangles and squares indicate putative cytosolic, peroxisomal and plastidial monomeric Cu,Zn-SOD forms, respectively. Black arrows point out other cross-reactive unknown proteins. (JPG 1719 kb)

Additional file 3: **Table S2.** Identification of olive (cv. ‘Picual’) pollen OeCSD1.1A (splicing A form) and OeCSD1.1B (splicing B form) recombinant proteins by MALDI-TOF/TOF analysis. (PDF 236 kb)

Additional file 4: **Figure S2.** Superoxide dismutase (SOD) activity and native 1-D immunodetection assays of olive pollen SODs. Total SOD activity (right panel) and the corresponding native immunoblot (left panel) from the OeCSD1.1A (splicing A form; accession no. EU250770.1) and OeCSD1.1B (splicing B form; EU250769.1) recombinant proteins, as well as from an olive pollen (cv. ‘Picual’) protein extract. Single bands of SOD activity associated to the recombinant proteins and to the pollen extract (I-VII) are visible in the activity gel (right panel). In parallel, the anti-Cu,Zn-SOD antibody reacts with similar bands in the immunoblot (left panel). (JPG 1628 kb)

Additional file 5: **Figure S3.** Olive (cv. ‘Picual’) pollen Cu,Zn-SOD amino acid sequences alignment obtained using Clustal W software (http://www.ebi.ac.uk/Tools/clustalw/). Sequences were obtained in our lab from cDNA and deposited on GenBank database. Post-translational modifications were predicted by using the ScanProsite software (http://prosite.expasy.org/scanprosite/). Dashed, dotted and solid line boxes correspond to putative glycosylation, casein kinase phosphorylation, and protein kinase C phosphorylation sites, respectively. Shadowed boxes indicate the SOD consensus motifs. Filled arrowheads point at His residues involved in the Cu2+ binding site, taking part in the dismutation reaction. Empty arrowheads point at the amino acid residues involved in the Zn2+ binding site, aimed to stabilize the enzyme. Cys56 and Cys145 taking part of a disulfide bond are also indicated. Amino acid sequences of OeCSD1.1A (accession no. EU250770.1) and OeCSD1.1B (EU250769.1) proteins, which were used to generate the recombinant proteins, are indicated in bold. Group A contains the following redundant sequences (GenBank accession no.): EU250759, EU250765, EU250768, EU250770, EU250790, EU250786, EU250788, EU250789, EU250758, EU250761, EU250764, EU250766, EU250771, EU250774, EU250776, EU250778, EU250781, EU250783, EU250791, EU250793, EU250775, EU250785. Group B contains the following redundant sequences (Genbank accession no.): EU250760, EU250772, EU250792, EU250796. (JPG 869 kb)

Additional file 6: **Figure S4.** Construct maps SODc-pKB6 (5678 bp) and SODd-pKB6 (5654 bp) for protein expression of the two olive pollen sequences representative of the complete (accession no. EU250770.1) and deleted (EU250769.1) forms of a cytosolic Cu,Zn-SOD, respectively. Sequences were cloned in frame into the pKB6 vector (Rekom Biotech SL, Granada, Spain) using the BamHI y HindIII sites. The position of other restriction sites, and key signatures of the T7-P lac operon and kanamycin resistance genes are also displayed. (JPG 3265 kb)

## Abbreviations

NO: Nitric oxide
ROS: Reactive oxygen species
SOD: Superoxide dismutase
