# Supplementary material for: Identification of novel superoxide dismutase isoenzymes in the olive (Olea europaea L.) pollen
Source: BMC Plant Biol. 2018 Jun 8;18:114. doi: 10.1186/s12870-018-1328-z (PMC5994013; doi:10.1186/s12870-018-1328-z)
Supplement: Supplementary file 1 — Table S1. Identification of protein bands (I-VII) from SOD activity native gels (cv. ‘Picual’) by nano-LC-ESI-MS/MS analysis. (PDF 238 kb) [file 12870_2018_1328_MOESM1_ESM.pdf]

Table S1. Identification of protein bands (I-VII) from SOD activity native gels (cv. 'Picual') by nano-LC-ESI-MS/MS analysis.

Gel band	Identified protein (Species)	Accession no.	Coverage (%)	Score[a]	m/z meas.	Δ m/z [ppm]	Range	Peptides
I	Superoxide dismutase [Mn], mitochondrial (*Hevea brasiliensis*)	P35017	11.2	95	1417.67<br>1626.88	-7.27<br>-1.70	59-69<br>167-181	K.HHQTYITNYNK.A<br>K.LVVETTANQDPLVTK.G
II	Superoxide dismutase [Cu-Zn] 2 (*Olea europaea*)	Q8L5E0-1	17	78	1378.68	-4.72<br>-20.81	102-114<br>115-127	K.QIPLTGPHSIIGR.A<br>R.AVVVHSDPDDLGR.G
III	Superoxide dismutase [Mn], mitochondrial (*Pisum sativum*)	P27084	4.7	32	1417.67	2.62	68-78	K.HHQTYITNYNK.A
	Superoxide dismutase [Mn], mitochondrial (*Hevea brasiliensis*)	P35017	4.7	32	1417.67	-4.64	68-78	K.HHQTYITNYNK.A
IV	Superoxide dismutase [Cu-Zn] 2 (*Olea europaea*)	Q8L5E0-1	17	78	1378.68	-4.72<br>-20.81	102-114<br>115-127	K.QIPLTGPHSIIGR.A<br>R.AVVVHSDPDDLGR.G
VII	Superoxide dismutase [Cu-Zn] 2 (*Olea europaea*)	Q8L5E0-1	8.6	57	1387.79	-21.91	102-114	K.QIPLTGPHSIIGR.A
	Superoxide dismutase [Cu-Zn] (*Paulownia kawakamii*)	O49073	8.6	57	1387.79	-21.91	102-114	K.QIPLTGPHSIIGR.A

[a] Protein scores greater than 31 are significant ($p<0.05$). Protein score is -10*Log(P), where P is the probability that the observed match is a random event.
