# Supplementary figures and images for: Identification of novel superoxide dismutase isoenzymes in the olive (Olea europaea L.) pollen
Source: BMC Plant Biol. 2018 Jun 8;18:114. doi: 10.1186/s12870-018-1328-z (PMC5994013; doi:10.1186/s12870-018-1328-z)

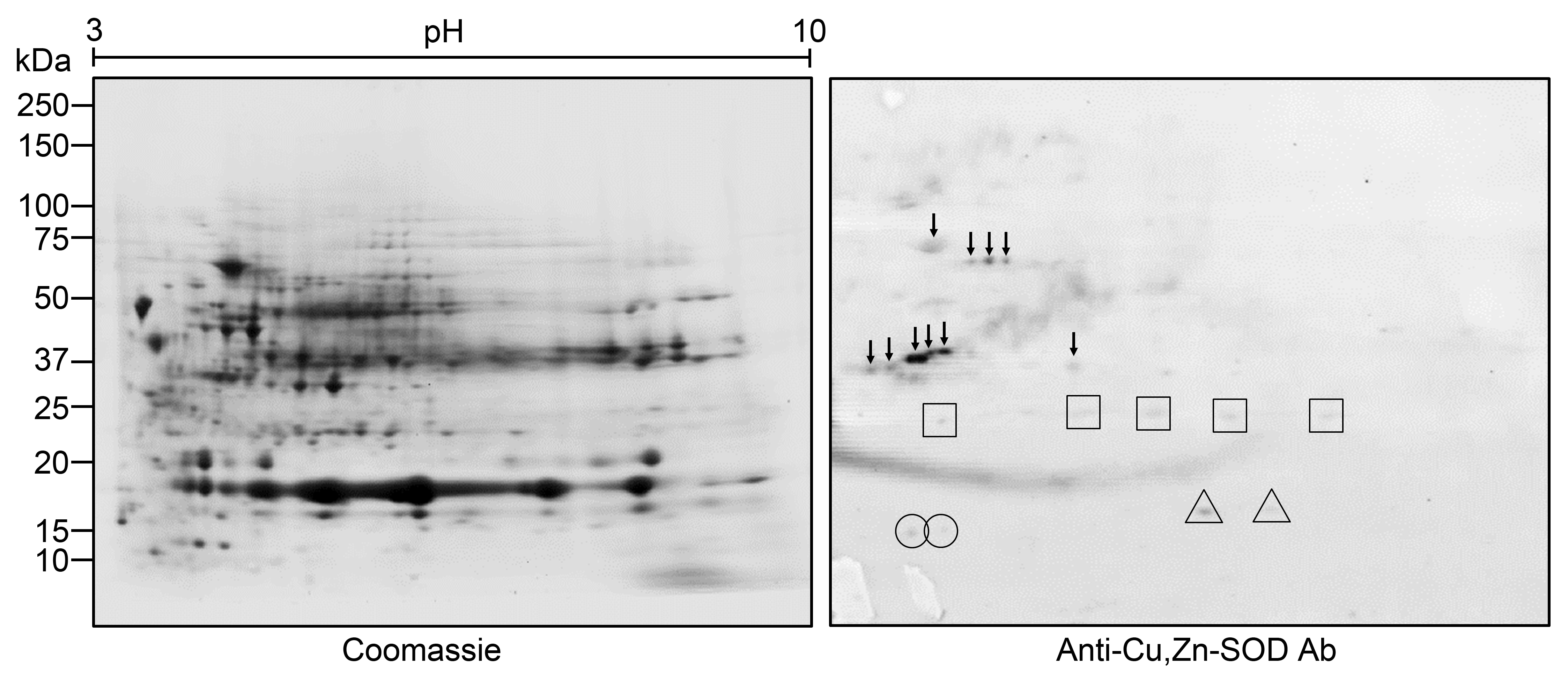

Supplement: Supplementary file 2 — Figure S1. Immunodetection of Cu,Zn-SOD enzymes in olive (cv. ‘Picual’) pollen protein extracts by 2-D Western blotting. Coomassie brilliant blue (CBB)-stained gel (left) and immunoblot probed with an anti-olive Cu,Zn-SOD antibody (right). Two hundred micrograms of total protein were loaded on the IEF strips. Protein markers are shown on the left. Circles, triangles and squares indicate putative cytosolic, peroxisomal and plastidial monomeric Cu,Zn-SOD forms, respectively. Black arrows point out other cross-reactive unknown proteins. (JPG 1719 kb) [file 12870_2018_1328_MOESM2_ESM.jpg]

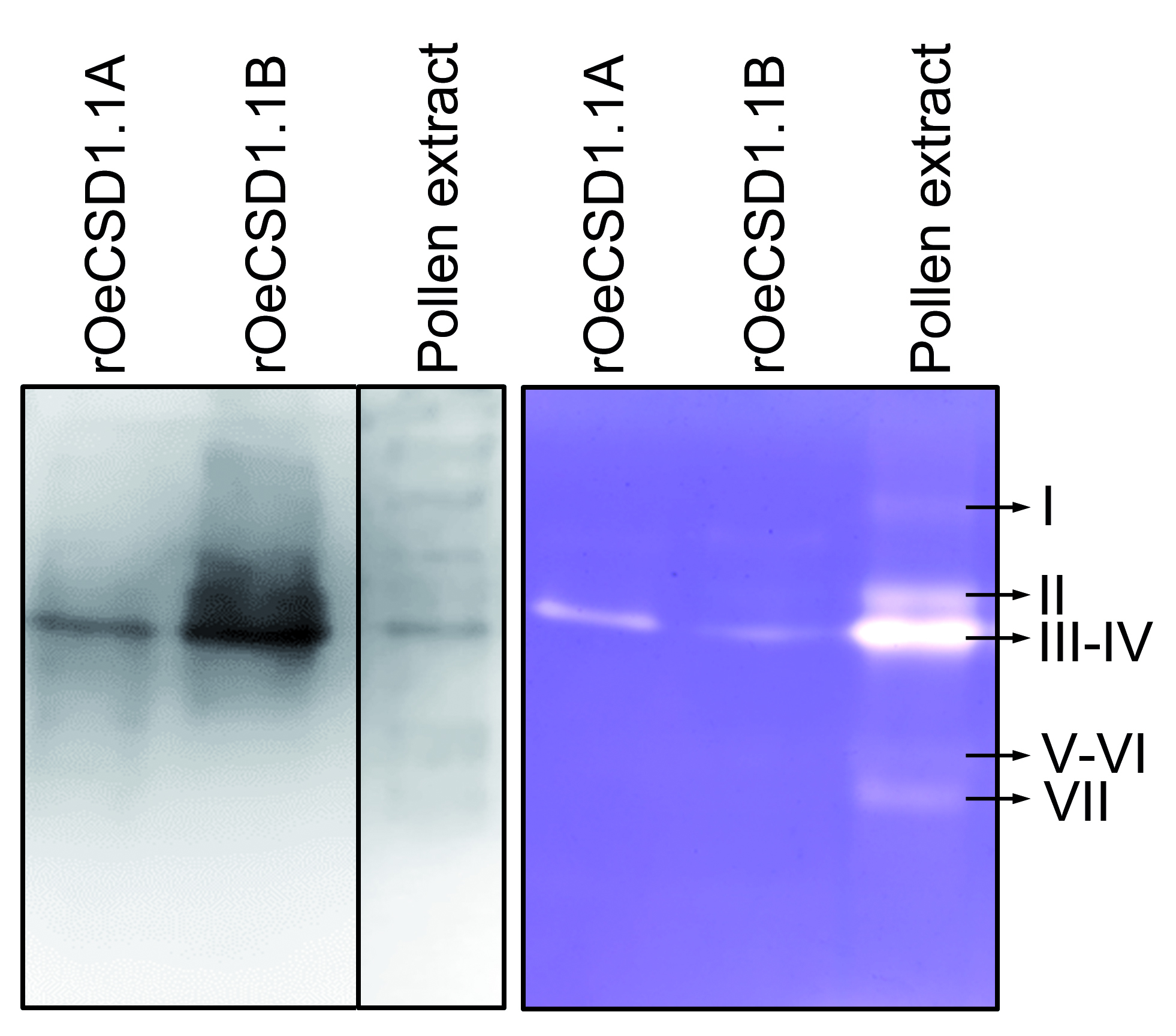

Supplement: Supplementary file 4 — Figure S2. Superoxide dismutase (SOD) activity and native 1-D immunodetection assays of olive pollen SODs. Total SOD activity (right panel) and the corresponding native immunoblot (left panel) from the OeCSD1.1A (splicing A form; accession no. EU250770.1) and OeCSD1.1B (splicing B form; EU250769.1) recombinant proteins, as well as from an olive pollen (cv. ‘Picual’) protein extract. Single bands of SOD activity associated to the recombinant proteins and to the pollen extract (I-VII) are visible in the activity gel (right panel). In parallel, the anti-Cu,Zn-SOD antibody reacts with similar bands in the immunoblot (left panel). (JPG 1628 kb) [file 12870_2018_1328_MOESM4_ESM.jpg]

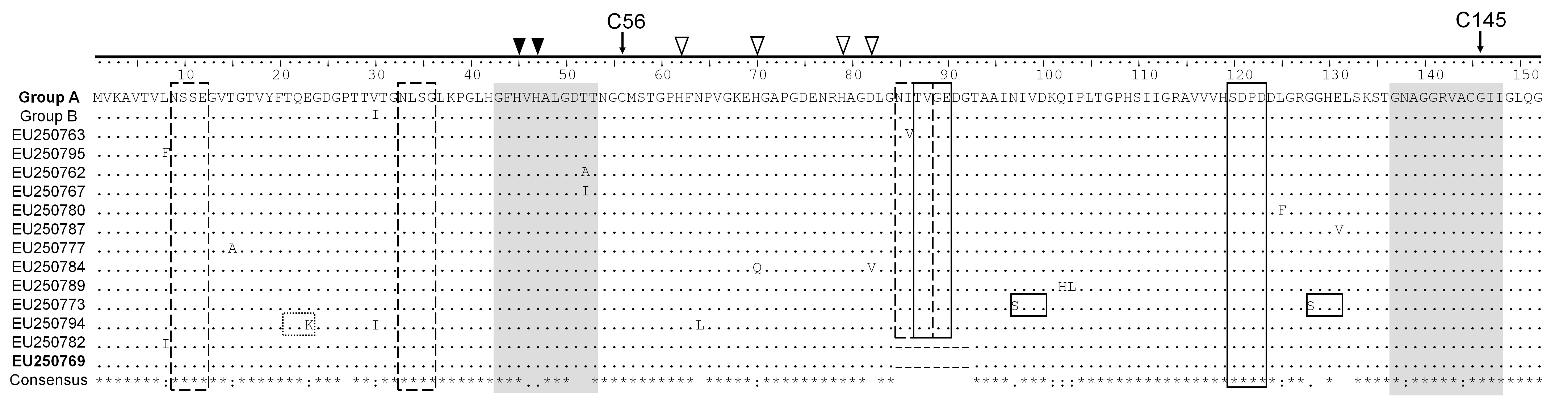

Supplement: Supplementary file 5 — Figure S3. Olive (cv. ‘Picual’) pollen Cu,Zn-SOD amino acid sequences alignment obtained using Clustal W software (http://www.ebi.ac.uk/Tools/clustalw/). Sequences were obtained in our lab from cDNA and deposited on GenBank database. Post-translational modifications were predicted by using the ScanProsite software (http://prosite.expasy.org/scanprosite/). Dashed, dotted and solid line boxes correspond to putative glycosylation, casein kinase phosphorylation, and protein kinase C phosphorylation sites, respectively. Shadowed boxes indicate the SOD consensus motifs. Filled arrowheads point at His residues involved in the Cu2+ binding site, taking part in the dismutation reaction. Empty arrowheads point at the amino acid residues involved in the Zn2+ binding site, aimed to stabilize the enzyme. Cys56 and Cys145 taking part of a disulfide bond are also indicated. Amino acid sequences of OeCSD1.1A (accession no. EU250770.1) and OeCSD1.1B (EU250769.1) proteins, which were used to generate the recombinant proteins, are indicated in bold. Group A contains the following redundant sequences (GenBank accession no.): EU250759, EU250765, EU250768, EU250770, EU250790, EU250786, EU250788, EU250789, EU250758, EU250761, EU250764, EU250766, EU250771, EU250774, EU250776, EU250778, EU250781, EU250783, EU250791, EU250793, EU250775, EU250785. Group B contains the following redundant sequences (Genbank accession no.): EU250760, EU250772, EU250792, EU250796. (JPG 869 kb) [file 12870_2018_1328_MOESM5_ESM.jpg]

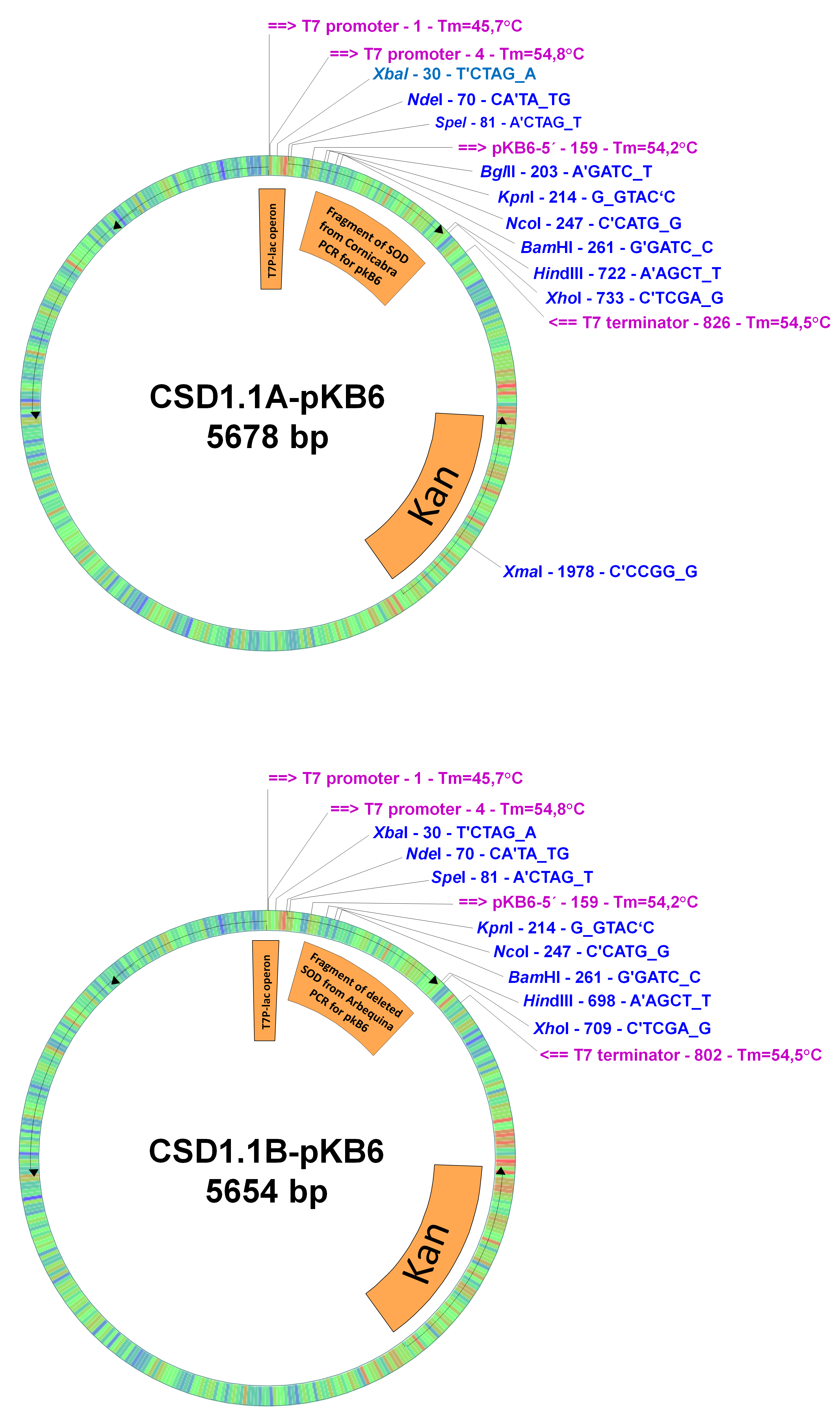

Supplement: Supplementary file 6 — Figure S4. Construct maps SODc-pKB6 (5678 bp) and SODd-pKB6 (5654 bp) for protein expression of the two olive pollen sequences representative of the complete (accession no. EU250770.1) and deleted (EU250769.1) forms of a cytosolic Cu,Zn-SOD, respectively. Sequences were cloned in frame into the pKB6 vector (Rekom Biotech SL, Granada, Spain) using the BamHI y HindIII sites. The position of other restriction sites, and key signatures of the T7-P lac operon and kanamycin resistance genes are also displayed. (JPG 3265 kb) [file 12870_2018_1328_MOESM6_ESM.jpg]
