# Supplementary material for: Identification of novel superoxide dismutase isoenzymes in the olive (Olea europaea L.) pollen
Source: BMC Plant Biol. 2018 Jun 8;18:114. doi: 10.1186/s12870-018-1328-z (PMC5994013; doi:10.1186/s12870-018-1328-z)
Supplement: Supplementary file 3 — Table S2. Identification of olive (cv. ‘Picual’) pollen OeCSD1.1A (splicing A form) and OeCSD1.1B (splicing B form) recombinant proteins by MALDI-TOF/TOF analysis. (PDF 236 kb) [file 12870_2018_1328_MOESM3_ESM.pdf]

Table S2. Identification of olive (cv. 'Picual') pollen OeCSD1.1A (splicing A form) and OeCSD1.1B (splicing B form) recombinant proteins by MALDI-TOF/TOF analysis.

Name	Identified protein (Species)	Accession no. [a]	Coverage (%)	Score [b]	m/z meas.	Δ m/z [ppm]	Range	Peptides
rOeCSD1.1A	Superoxide dismutase [Cu,Zn] 2 (*Olea europaea*)	Q8L5E0-1	48.7	83	1081.4452	-18.97	69-78	K.EHGAPGDENR.H
					2280.1236	-10.08	79-101	R.HAGDLGNITVGEDGTAAINIVDK.Q
					1388.7845	-11.80	102-114	K.QIPLTGPHSIIGR.A
					1379.6722	-13.87	115-127	K.AVVVHSDPDDLGR.G
					2088.0330	-6.656	115-134	K.AVVVHSDPDDLGRGGHELSK.S
					719.3377	-7.561	135-142	K.STGNAGGR.V
rOeCSD1.1B	Superoxide dismutase [Cu,Zn] 2 (*Olea europaea*)	Q8L5E0-2	46.5	107	1081.4414	-22.46	69-78	K.EHGAPGDENR.H
					1494.7627	-18.97	79-93	R.HAGDLGTAAINIVDK.Q
					1388.7796	-15.32	94-106	K.QIPLTGPHSIIGR.A
					1379.6650	-19.13	107-119	K.AVVVHSDPDDLGR.G
					719.3218	-29.64	127-134	K.STGNAGGR.V

[a] Uniprot database accesion number.

[b] Protein scores greater than 16 are significant ($p<0.05$). Protein score is -10*Log(P), where P is the probability that the observed match is a random event.
